# Microbial biodiversity in Tunisian olive grove soils: a reservoir of phytopathogenic fungi and potential beneficial microorganisms

**DOI:** 10.3389/ffunb.2026.1770745

**Published:** 2026-03-16

**Authors:** Meryam Belhedi, Besma Sghaier-Hammami, Sofienne B. M. Hammami, Samar Ben Slema, Palmira De Bellis, Stefania Somma, Haithem Nafati, Khaled Hibar, Charlie Abi Saad, Antonio Moretti, Mario Masiello

**Affiliations:** 1 Horticultural Sciences Laboratory, LR13AGR01, National Agronomic Institute of Tunisia, University of Carthage, Tunis, Tunisia; 2 Laboratory of Bioggressors and Integrated Pest Management in Agriculture, LR14AGR02, National Agronomic Institute of Tunisia, University of Carthage, Tunis, Tunisia; 3 Institute of Sciences of Food Production, National Research Council, Bari, Italy; 4 Département Physique, Faculté des Sciences de Tunis, Tunis, Tunisia; 5 Olive tree Institute, Laboratory of Integrated Olive Production in the Humid, Sub-humid and Semi-arid Region (LR16IO3), Sousse, Tunisia; 6 The Department of Soil, Plant and Food Sciences (DISSPA), University of Bari Aldo Moro, Bari, Italy

**Keywords:** *Bacillus* spp., intercropping system, olive tree (*Olea europaea*), potential biocontrol agents, rhizosphere microbiome, soil-borne pathogen, wilting disease

## Abstract

**Introduction:**

Intercropping in olive orchards increases the risk of soil-borne fungal infections, particularly when associated crops are susceptible to the same pathogens. This study aimed to identify soil-borne microorganisms colonizing the roots and rhizosphere of olive trees in Tunisia intercropped with *Solanaceae* plants and to evaluate co-occurring bacterial communities for their potential to mitigate wilt disease and promote plant health.

**Material and methods:**

Endophytic fungi and bacteria were isolated from olive soils and roots collected from three olive orchards subjected to different intercropping systems. Fungal strains were molecularly identified at the species level using Internal Transcribed Spacer (ITS) and translation elongation factor 1-α (*TEF1*) gene sequencing, while bacterial strains were characterized by rep-PCR profiling and 16S rDNA sequencing. The pathogenicity of selected *Fusarium* strains was assessed by *in vitro* inoculation of detached olive leaves, olive twigs, and tomato seedlings. Antagonistic activity of bacterial strains against selected *Fusarium* species was evaluated using dual-culture assays, and bacteria–fungi interactions were further investigated by scanning electron microscopy (SEM).

**Results and discussion:**

A total of 83 fungal and 40 bacterial strains were isolated. The fungal community was dominated by *Fusarium* species (62%), followed by *Phoma* (13%) and *Alternaria* (10%) species, while *Verticillium dahliae* was not detected at any site. The prevalence and virulence of *Fusarium* varied among olive groves, with the highest incidence observed at Sidi Bou Ali, where olive trees were intercropped with tomato, and the lowest at Kairouan, where potato intercropping was less frequent. Pathogenicity assays showed that 12 out of 15 of the tested *Fusarium* strains caused symptoms on both olive tissues and tomato seedlings. Bacterial communities were dominated by *Bacillus* species and *Priestia megaterium*. *Bacillus* species were particularly abundant at the site with the highest *Fusarium* pressure. The *in vitro* assay showed that several bacteria exhibited antagonistic activity against pathogenic fungi, with growth inhibition ranging from 8% to 68%, including volatile organic compound–mediated effects. SEM analyses revealed that *Bacillus amyloliquefaciens* inhibited fungal growth through biofilm formation and hyphal alteration.

## 1 Introduction

Olive (*Olea europaea* L.), one of the oldest domesticated fruit trees, represents an important crop in the Mediterranean Basin, accounting for almost 90% of the olive cultivation area worldwide. Tunisia alone cultivates about 1.9 million hectares and contributes around 7% of global olive oil production (FAOSTAT, 2024). Olive groves represent a relatively stable agroecosystem due to the ability of olive trees to adapt to a wide range of environmental conditions. Their resilience is also supported by the wide occurrence of beneficial microorganisms in the soil where they grow, which enhance tolerance to biotic and abiotic stresses, especially those occurring in the rhizosphere (Fernández-González et al., 2019). However, the rhizosphere of olive groves is threatened by several soil-borne pathogens, among which *Verticillium dahliae* is the most frequently reported and is considered the most important fungal pathogen affecting olive trees (Jiménez-Díaz et al., 2012; Keykhasaber et al., 2018; López-Escudero and Mercado-Blanco, 2011). In recent years, *Fusarium* species have emerged as additional pathogens associated with olive dieback in several countries worldwide (Nicoletti et al., 2020), including Morocco (Chliyeh et al., 2014), Spain (Hernández et al., 1998), Greece (Markakis et al., 2021), and Tunisia (Belhedi et al., 2025; Gharbi et al., 2020; Trabelsi et al., 2017). This rise can be attributed to changes in olive cultivation methods, the expansion of new plantations into *Fusarium*-infected areas, and the use of infected planting material. Indeed, the growing global demand for olive oil is leading to planting new olive orchards in the Mediterranean regions and a shift from traditional rainfed agriculture to high-density system (Cardoni and Mercado-Blanco, 2023). The new agronomic practices, together with climate changes, could drive the spread of different soil-borne pathogens, the incidence and the severity of olive diseases (Benítez-Cabello et al., 2023; Rallo et al., 2013). In addition, the cultivation of *Solanaceae* plants (e.g., tomato, pepper, eggplant) alongside olive has been reported to enhance the persistence of soil-borne fungi by providing alternative susceptible hosts (Gharbi et al., 2020; Nigro et al., 2005). Such intercropping practices may facilitate pathogen adaptation and transfer from annual to perennial hosts, thereby intensifying disease pressure in olive orchards. In Tunisia, however, this interaction remains poorly investigated, and the role of intercropped *Solanaceae* plants on olive tree diseases is still unclear.

Olive disease management relies mainly on agronomic measures, including the use of certified planting material, appropriate pruning, and improved soil and irrigation management (Fernández-Escobar et al., 2013). Yet, intensive systems and heavy agrochemical use can disrupt the natural soil microbiome (Bizos et al., 2020; Montes-Osuna and Mercado-Blanco, 2020; Zipori et al., 2021). Beneficial microbial species, belonging to *Bacillus*, *Pseudomonas*, *Paenibacillus*, *Trichoderma*, and *Glomus* genera, play crucial roles in plant health by promoting growth, enhancing nutrient uptake, and suppressing pathogens (Aloo et al., 2019; Mercado-Blanco and Lugtenberg, 2014). For instance, *Bacillus velezensis* and some *Pseudomonas* species isolated from olive rhizospheres inhibit *Fusarium* spp. and *V. dahliae*, through biofilm formation and production of siderophores (Cheffi et al., 2019; Mercado-Blanco et al., 2004).

A comprehensive knowledge of microbial communities associated to the olive root system, including the root endosphere and the rhizosphere soil, as well as knowledge on the influence of biotic and abiotic factors on these communities is crucial for selecting specific fungi and bacteria that enhance olive traits under specific conditions. This study aimed to provide a picture of the microbial communities of olive groves intercropped with *Solanaceae* plants in Tunisia, to characterize fungal pathogens isolated from the roots of olive trees showing wilt symptoms, and to select beneficial microorganisms with potential biocontrol properties.

## 2 Materials and methods

### 2.1 Origin of the samples and microorganisms’ isolation

Three different olive groves (Chemlali *cultivar*), located in two different regions and characterized by different intercropping systems, were considered. Site 1, located in Kairouan region, included forty-year-old olive trees intercropped with potato plants (*Solanum tuberosum* L.), continuously cultivated in this field for seven crop seasons. Site 2, located in Hergla, in Sousse region, consisted of ten-year-old olive trees intercropped with potato plants, cultivated in this field for thirty consecutive years. Site 3, located in Sidi Bou Ali, in Sousse region, included young olive trees intercropped with tomato (*Solanum lycopersicum* L.) and pepper (*Capsicum annuum* L.). At each site, four parcels were selected, and five trees were sampled per parcel (20 trees per site), with each tree considered as one biological replicate. Sampling was conducted after the harvest of the intercropped crops. For each tree, approximately 1 L of soil containing roots was collected at the projection of the canopy edge (drip line), which corresponds to the main rhizosphere interaction zone between olive tree roots and the associated intercropped vegetation. This zone was deliberately chosen to characterize microbial communities shaped by intercropping practices and root–root interactions. Soil samples were collected from four equidistant points around each tree and pooled to generate one composite sample per tree. Roots were subsequently separated from the soil by sieving. Sampling followed a completely randomized design arranged along a Z-shaped transect within each parcel.

After washing with running tap water, portions of roots were surface disinfected by dipping in 2% sodium hypochlorite solution for 2 minutes, followed by three rinses in sterile distilled water. Small pieces of root of about 3 mm diameter were transferred on Potato Dextrose Agar (PDA) and Malt Extract Agar (MEA), both amended with streptomycin sulphate (100 mg L^-1^) and neomycin sulphate (50 mg L^-1^). For each soil sample, 1.5 g was transferred to a sterile microbiological tube containing 13.5 mL of sterile distilled water and shaken for 30 min at 150 rpm in an orbital shaker. Aliquots (100 µL) of three serial dilutions were spread on Plate Count Agar (PCA). All plates were incubated at 25 ± 1°C for 5 days under an alternating 12-hour light/dark cycle. After incubation, fungal and bacterial colonies, selected on the basis of their morphological characteristics, were purified for further analysis. Each fungal colony was purified by spreading conidial suspension on Water Agar (WA) and, after 18 hours of incubation at 25 ± 1°C, a single germinated spore was collected under dissecting microscope and transferred on new PDA plate. To obtain pure bacterial cultures, single colonies were picked up and purified by repeated streaking on PCA medium.

### 2.2 Molecular identification of isolated microorganisms

#### 2.2.1 Fungal species identification

Mycelium of 3-day-old colonies, grown on sterilized cellophane disks overlaid on PDA plates, was collected by scraping. Genomic DNA was extracted and purified from powdered lyophilized mycelia (each sample, 10–15 mg) using the Wizard Magnetic DNA Purification System for Food kit (Promega, Madison, WI, USA), according to the manufacture’s protocol. The quantity and integrity of DNA were checked by comparison with a standard 1 kb DNA Ladder (Fermentas GmbH, St. Leon-Rot, Germany) on 0.8% agarose gel, after electrophoretic separation.

For each selected strain, an informative fragment of the Internal Transcribed Spacer (ITS) was amplified and sequenced using the primer pair ITS-4/ITS-5 (White et al., 1990). In addition, all fungal strains belonging to *Fusarium* genus, were molecularly characterized by sequencing the translation elongation factor 1-α (*TEF1*) gene, commonly reported as the most informative gene for *Fusarium* identification to species level (Geiser et al., 2004), using the primer pair EF1/EF2 (O’Donnell et al., 1998). The Polymerase Chain Reaction mixture (15 μL) contained 30 ng of genomic DNA, 0.45 μL of each primer (10 µM), 1.2 μL of dNTPs (10mM), 1.5 µL of 10x buffer and 0.125 μL of Hot Start Taq DNA Polymerase (Fisher Molecular Biology, 1 U μL^-1^, Rome, Italy). The amplifications were carried out in a Mastercycler EP Gradient thermal cycler (Eppendorf, Hamburg, Germany). ITS region was amplified using the following conditions: initial denaturation of 50 sec at 72°C, followed by 40 cycles of 30 sec at 95°C, 30 sec at 52°C, 50 sec at 72°C, and a final extension of 5 min at 72°C. The *TEF1* fragment gene was amplified using the following parameters: initial denaturation of 2 min at 95°C, followed by 35 cycles of 50 sec at 95°C, 50 sec at 59°C, 1 min at 72°C, and a final extension of 7 min at 72°C.

The PCR products, stained with GelRed^®^ (Biotium Inc., Fremont, CA, USA), were checked after electrophoretic separation on 1.5% agarose gel in 1X TAE buffer under UV light by comparison with a 100 bp DNA Ladder (ThermoFisher Scientific, Waltham, Massachusetts, USA). Before sequencing, the PCR products were purified with an enzymatic EXO/FastAP mixture (Exonuclease I and FastAP thermosensitive alkaline phosphatase, ThermoFisher Scientific, Waltham, Massachusetts, USA). Sequence reactions were performed for both strands using a BigDye Terminator v3.1 Cycle Sequencing Ready Reaction Kit (Applied Biosystems, Foster City, CA, USA) according to the manufacturer’s recommendations. The labeled products were purified by filtration through Sephadex G-50 (5%) (Sigma-Aldrich, Saint Louis, MO, USA) and analyzed using an ABI PRISM 3730 Genetic Analyzer (ThermoFisher Scientific, Waltham, Massachusetts, USA). The FASTA sequences obtained were analyzed and assembled using BioNumerics v. 5.1 software (Applied Maths, Kortrijk, Belgium). For molecular identification at the genus or species level, the ITS obtained sequences were compared with the sequences available in the NCBI Database through the BLASTN program.

Nucleotide sequences of *TEF1* gene, joined to sequences of species reference strains retrieved from GenBank, were aligned using the ClustalW algorithm (Thompson et al., 1994). Phylogenetic relationships were studied using the maximum likelihood method with MEGA software version 7 (Kumar et al., 2016). The bootstrap analyses (Felsenstein, 1985) were conducted to determine the confidence of internal nodes using a heuristic search with 1000 replicates, removing gaps. The field strain MB38, identified as *Clonostachys rosea*, was used as outgroup taxon.

#### 2.2.2 Bacterial species identification

Genomic DNA was extracted using the Wizard Genomic DNA Purification Kit (Promega, Madison, WI, USA) following the manufacturer’s protocol. All isolated bacterial strains were molecularly characterized through rep-PCR assay by using the primer pair REP-1R-Dt (5 –IIINCGNCGNCATCNGGC–3) and REP-2R-Dt (5 –NCGNCTTATCNGGCCTAC– 3) (Hyytiä-Trees et al., 1999). The PCR reactions were carried out in a total volume of 25 μl, containing 23 μL of Mega Mix (Microzone Ltd., United Kingdom), 0.5 μL of each primer (10 mM) and 1 μl of genomic DNA (15 ng). Amplifications were performed in a Mastercycler EP Gradient thermal cycler (Eppendorf, Hamburg, Germany) as reported by Zicca et al. (2020). Amplicons were separated by microfluidic electrophoresis using the DNA7500 LabChip kit with the Agilent 2100 Bioanalyzer (Agilent Technologies, Waldbronn, Germany), according to the manufacturer’s instructions. The rep-PCR profiles were analyzed and compared using the software provided by the same company.

All bacterial strains were grouped on the basis of their rep-PCR profile and for each different profile, a representative strain was identified by 16S rRNA gene sequencing using the universal bacterial primers 27f-YM and 1492r (Frank et al., 2008). PCR reactions were carried out in a 50 μl mixture containing 5 μl of 10× AccuPrime™ Pfx Reaction Mix, 1.25 U of AccuPrime™ Pfx DNA polymerase (Thermo Fisher Scientific, Waltham, Massachusetts, USA), 0.3 μM of each primer, and 1 μl of genomic DNA. The amplification program consisted of an initial denaturation at 95°C for 2 min, followed by 35 cycles of denaturation at 95°C for 30 s, annealing at 60°C for 5 cycles, 55°C for 5 cycles, and 48°C for 25 cycles, with extension at 68°C for 3 min, and a final extension at 68°C for 10 min. PCR products were sequenced using the BigDye™ Terminator cycle sequencing kit on an ABI Prism 3730xl DNA Analyzer (Thermo Fisher Scientific, Waltham, Massachusetts, USA). Sequences were assembled with BioNumerics v. 5.1 (Applied Maths, Austin, Texas, USA) and compared to type strain sequences available in the NCBI database (BLASTN, “Sequences from type material” option). Bacterial strains were tentatively assigned to a bacterial species on the basis of the highest score of the alignment and the percentage of identity between their 16S rRNA gene sequences and those of type strains in the database.

### 2.3 *In vitro* pathogenicity test

To assess the potential pathogenicity of the isolated fungal species on olive tree and *Solanaceae* plants, conidial suspensions of selected fungal strains, chosen based on molecular identification, were used to inoculate detached leaves and stems of olive tree seedlings, as well as one-month-old tomato seedlings. Olive leaves were placed in rectangular Petri dishes on moistened sterile paper. Aliquots of 20 µl of conidial suspension, adjusted to 10^6^ spores mL^-^¹, were injected into light incision made in the middle of each leaf. For olive stems, portions of about 110 mm and 25 mm in diameters were placed in Petri dishes. An incision, 10 mm deep, was made in the bark of each stem and inoculated with the same conidial suspension used for the leaves. A negative control, leaves and stems injected with sterile distilled water, was also considered. To assess the pathogenicity on *Solanaceae* plants, tomato seedlings were uprooted, their roots washed with tap water and then immersed for 4 days in the previously prepared conidial suspensions. Tomato seedlings immersed in sterile distilled water were used as negative control. All experiments were performed on five replicates.

After seven days of incubation at room temperature with a photoperiod of 12h, the number of olive leaves and stems and tomato seedlings showing symptoms on the total number of inoculated ones was recorded. Disease severity on olive and tomato leaves was visually assessed based on the extent of leaf yellowing and wilting, following the empirical scale: 0 = no visible symptoms; 1 = yellowing or wilting affecting up to 1/3 of the leaf; 2 = yellowing or wilting affecting 1/2 of the leaf; 3 = yellowing or wilting affecting 2/3 of the leaf; 4 = yellowing or wilting affecting the whole leaf; and 5 = complete necrosis or rolling of the leaf or leaf drop.

Disease Severity Index (DSI) was calculated using the formula:


$$DSI\;\left(\%\right)=\;\frac{\sum \left(n\times v\right)}{N\times V}\times 100$$

where n is the number of leaves in each severity category, v is the severity rating, N is the total number of assessed leaves, and V is the maximum severity rating. Pathogenicity on the stems was assessed by counting the number of infected stems on the total ones. A stem was considered infected when a visibly hollow surrounding the point of conidial suspension injection was observed.

### 2.4 *In vitro* antagonist assay using bacteria

The antagonistic activity of identified bacterial strains was evaluated against two *Fusarium* strains, MB1 (*F. brachygibbosum*) and MB54 (*F. oxysporum*). The capability of these bacteria to inhibit mycelial growth was assessed by a dual culture assay on PCA medium (Zhao et al., 2014). In detail, a 6-mm mycelial plug collected from the margin of actively growing colony of each *Fusarium* strain was placed at the center of the plate, and two drops (5 µL each) of the bacterial suspension (10^9^ CFU/mL) were inoculated 1 cm from the fungal plug. Control plates consisted of fungal plug without bacteria. All treatments were replicated three times and incubated for seven days at 25°C, under a photoperiod of 12h.

To evaluate the antagonistic activity, fungal colony diameters were measured every two days and used to calculate the percentage of mycelial growth inhibition.

The mycelial growth inhibition, expressed as percentage, was calculated using the following formula:


$$Mycelial\;growth\;inhibition\;\left(\%\right)=\;\frac{Dc-Dt}{Dc}\times 100$$

Where *Dc* is the mean value of the two orthogonal diameters of the control colony and *Dt* is the mean value of the two orthogonal diameters of the fungal colonies’ growth in presence of bacterial strains.

### 2.5 Bacterial–fungal interactions via volatile organic compounds (VOCs)

Bacterial strains were used to assess their capacity to produce VOCs, capable of inhibiting mycelial growth. According to the method reported by Wu et al. (2019), with some modifications, an overnight bacterial culture in TSB broth was spread onto a PDA plate. The lid was replaced by a base plate of PDA containing a 4 mm diameter agar plug from an actively growing fungal mycelium of *Fusarium*. The two PDA base plates were sealed together with Parafilm. Control sets were prepared without bacteria in the bottom plate. The diameters of the fungal colonies, on three replicates, were measured after 7 days incubation at 25°C and used to calculate the mycelial growth inhibition as reported above.

### 2.6 Microscopic and SEM observations

After seven days of incubation, the effect of the *Bacillus* bacteria on the mycelium of the tested fungal strains was examined under an optical microscope. For high-resolution imaging, a Thermo Scientific™ Quanta™ 250 FEI scanning electron microscope (SEM) equipped with an Energy-Dispersive X-ray Spectroscopy (EDX) system was used (Thermo Fisher Scientific Inc., Waltham, MA, USA). Scanning electron microscopy was used to visualize sample surfaces at micro- to nanometer resolution. The electron beam interacts with the material to generate signals that reveal surface morphology, while the integrated EDX enabled qualitative and quantitative elemental analysis. Analyses were carried out under low-vacuum conditions to preserve the integrity of biological and non-conductive samples. Imaging was performed at different magnifications and in low-voltage mode to reduce damage and improve penetration.

### 2.7 Statistical analysis

The data were analyzed using R version 4.3.2. A one-way analysis of variance (ANOVA) was performed to evaluate the effects of bacterial strains on fungal mycelial growth and separately, the effect of fungal strains on plant material in pathogenicity assays. Differences between means were assessed using Tukey’s test with a significance threshold of p< 0.05. Results were presented in tables as means ± standard error, with homogeneous groups indicated. For clustering and heatmap visualization, abundance data were log transformed to reduce the influence of highly abundant taxa, using the ‘pheatmap’ package in R.

## 3 Results

### 3.1 Fungal species occurring in olive roots and rhizospheres

**Figure 1:**
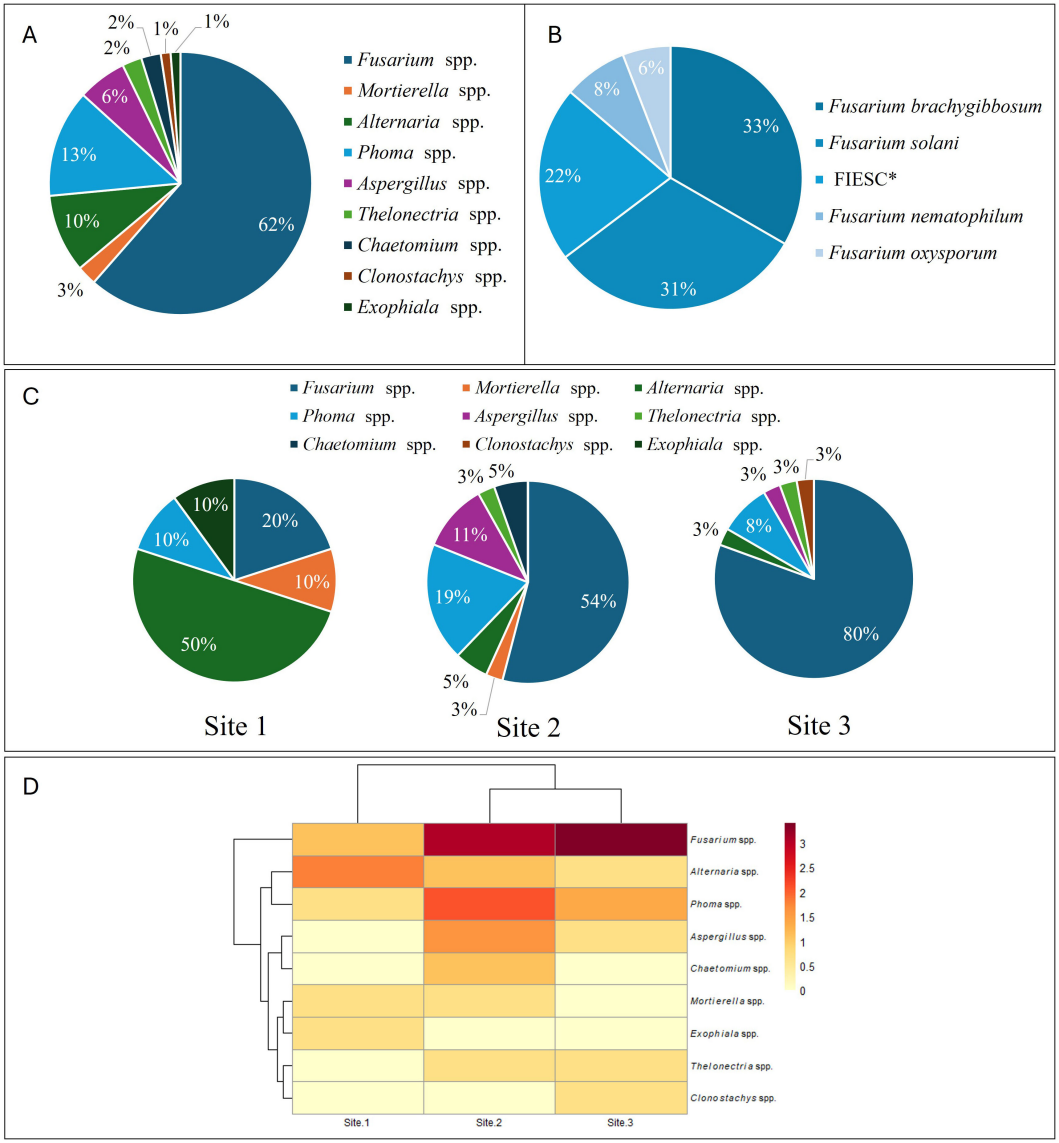
**(A)** Distribution of fungal genera, identified by ITS region sequencing from olive root–associated isolates pooled across all sampled olive orchards in Tunisia; **(B)** Distribution of Fusarium species, molecularly identified by ITS and *TEF1* gene sequencing on olive roots in Tunisia, representing the *Fusarium* spp. fraction shown in panel **(A)**, FIESC, *Fusarium incarnatum equiseti* Species Complex: *Fusarium longifundum*, *Fusarium clavum*, *Fusarium gracilipes*, *Fusarium caatingaense*; **(C)** Distribution of fungal isolates according to sampling site, site 1 (Kairouan), site 2 (Hergla, Sousse) and 3 (Sidi Bou Ali, Sousse). **(D)** Heat map of fungal strains abundance according to sampling site. The rows represent the isolated fungal strains, and the columns represent the sampling site. Color scale represents log-transformed abundance values.

Molecular identification of 83 fungal strains, based on ITS sequence analyses, revealed a great biodiversity of fungal species occurring in olive roots and rhizosphere, in Tunisia (**Supplementary Table 1**; **Figure 1A**). The majority of the strains (62%) belonged to *Fusarium* genus, followed by *Phoma* (13%) and *Alternaria* (10%) genera. At lesser extent, few strains identified as *Aspergillus*, *Chaetomium, Exophiala*, *Mortierella*, and *Thelonectria* were also identified (**Supplementary Table 1**, **Figure 1A**).

Molecular identification of *Fusarium* species strains, based on ITS and *TEF1* gene sequencing, revealed the presence of several *Fusarium* species on olive groves, in Tunisia, including *F. solani*, *F. nematophilum*, *F. oxysporum*, *F. brachygibbosum* and members of *Fusarium incarnatum-equiseti* Species Complex (FIESC), namely *F. caatingaense*, *F. clavum*, *F. gracilipes*. *F. incarnatum*, and *F. longifundum* (**Figure 1B**).

The fungal species distribution varied across the three sampling sites. In Site 1 (Kairouan), *Alternaria* genus was the most dominant (50% of the total isolated strains) followed by *Fusarium* (20%) and an even distribution of *Phoma*, *Exophiala* and *Mortierella* (10% each; **Figure 1C**). In Site 2 (Hergla, Sousse), *Fusarium* was the predominant genus (54%), followed by *Phoma* (19%) and *Aspergillus* (11%). In Site 3 (Sidi Bou Ali, Sousse), *Fusarium* was overwhelmingly dominant (80%), followed by *Phoma* (8%), whereas other fungal genera were detected to a very less extent (**Figure 1C**). Clustering analysis revealed closer similarities between the fungal composition of Hergla and Sidi Bou Ali compared with Kairouan, which showed a distinct profile (**Figure 1D**).

**Figure 2:**
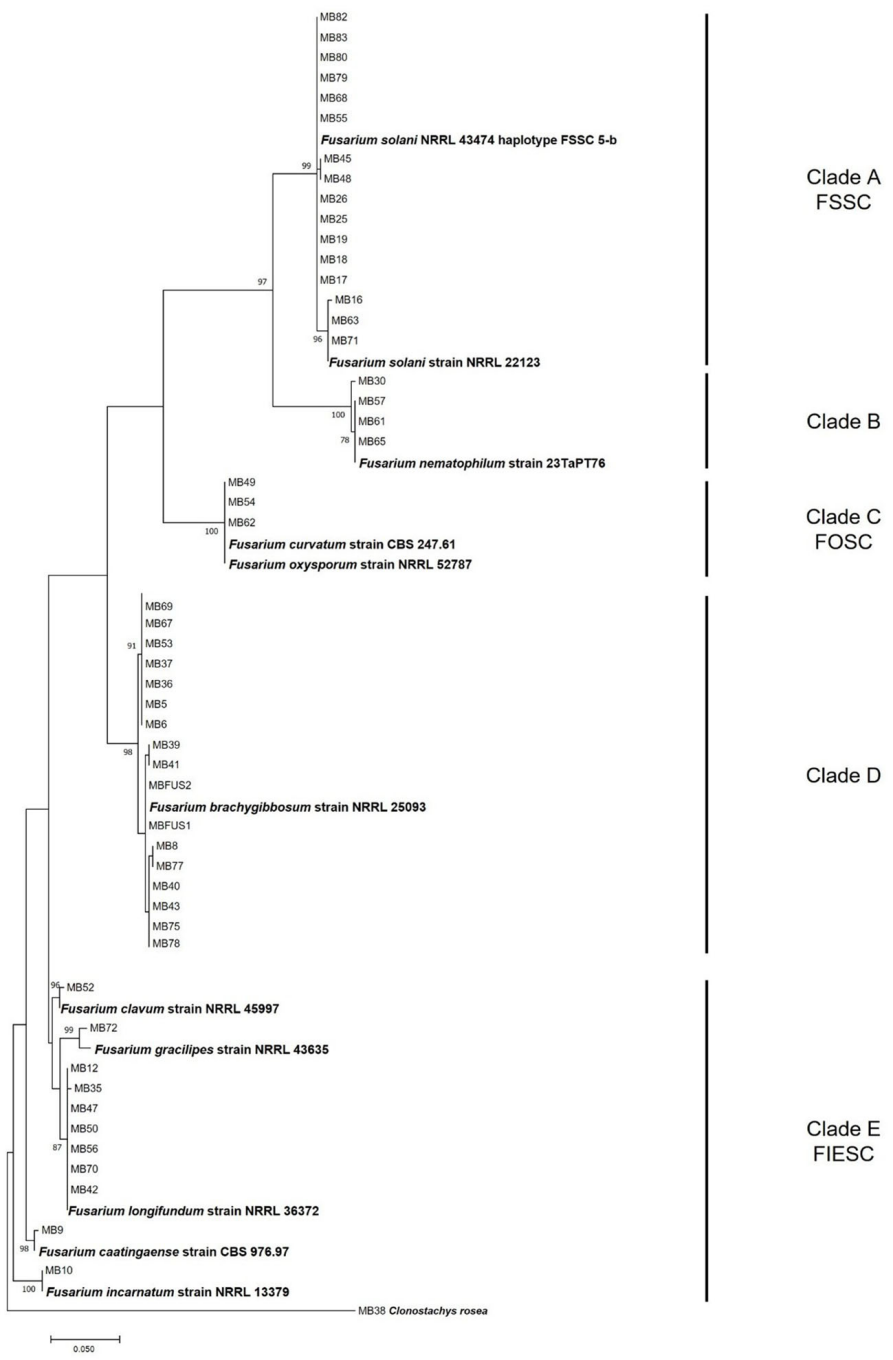
Phylogenetic tree generated by maximum likelihood method (bootstrap 1000 replicates) of *TEF1* gene sequences of 51 *Fusarium* strains isolated from olive roots, in Tunisia. Numbers on branches are bootstrap values (>70) based on 1000 replicates. FSSC: *Fusarium solani* Species Complex; FOSC: *Fusarium oxysporum* Species Complex; FIESC: *Fusarium incarnatum equiseti* Species Complex.

The phylogenetic analysis including the *TEF1* sequences of 51 *Fusarium* strains isolated from olive groves, 11 *Fusarium* reference sequences, and the sequence of *Clonostachys rosea* (MB38) used as an outgroup taxon, was shown in **Figure 2**. The phylogenetic tree was resolved in 5 well‐separated clades, supported by high bootstrap values. In clade A, sixteen strains are clustered together with the *F. solani* reference strains NRRL 22123 and NRRL 43474. In particular, three strains MB16, MB63 and MB71 showed high homology with *F. solani* NRRL 22123, while the other 13 *Fusarium* strains, showing 100% homology among them, grouped with *F. solani* NRRL 43474 haplotype FSSC 5-b. The strains MB30, MB57, MB61, and MB65 were identified as *F. nematophilum*, grouping with the reference strain 23TaPT76 (clade B). Only three strains, MB49, MB54 and MB62, grouping with the reference strains *F. oxysporum* NRRL 52787 and *F. curvatum* CBS 247.61, were identified as members of *Fusarium oxysporum* Species Complex (clade C, **Figure 2**).

The most abundant species, accounting for 17 strains out of 51, grouped together with *F. brachygibbosum* NRRL25093 reference stain (Clade D).

A great genetic biodiversity was observed in the clade E, including the members of FIESC (**Figure 2**). In this clade, a single strain, MB52, showed high homology with *F. clavum* NRRL 45997 reference strain; the strain MB72 grouped with *F. gracilipes* NRRL 43635 reference strain; seven strain showed 100% homology among them and with *F. longifundum* NRRL 36372 reference strain; two strains, MB9 and MB10, clustered together with the reference strains *F. caatingaense* CBS 976.97 and *F. incarnatum* NRRL 13379, respectively (**Figure 2**).

### 3.2 Diversity of bacterial communities in olive rhizosphere

**Figure 3:**
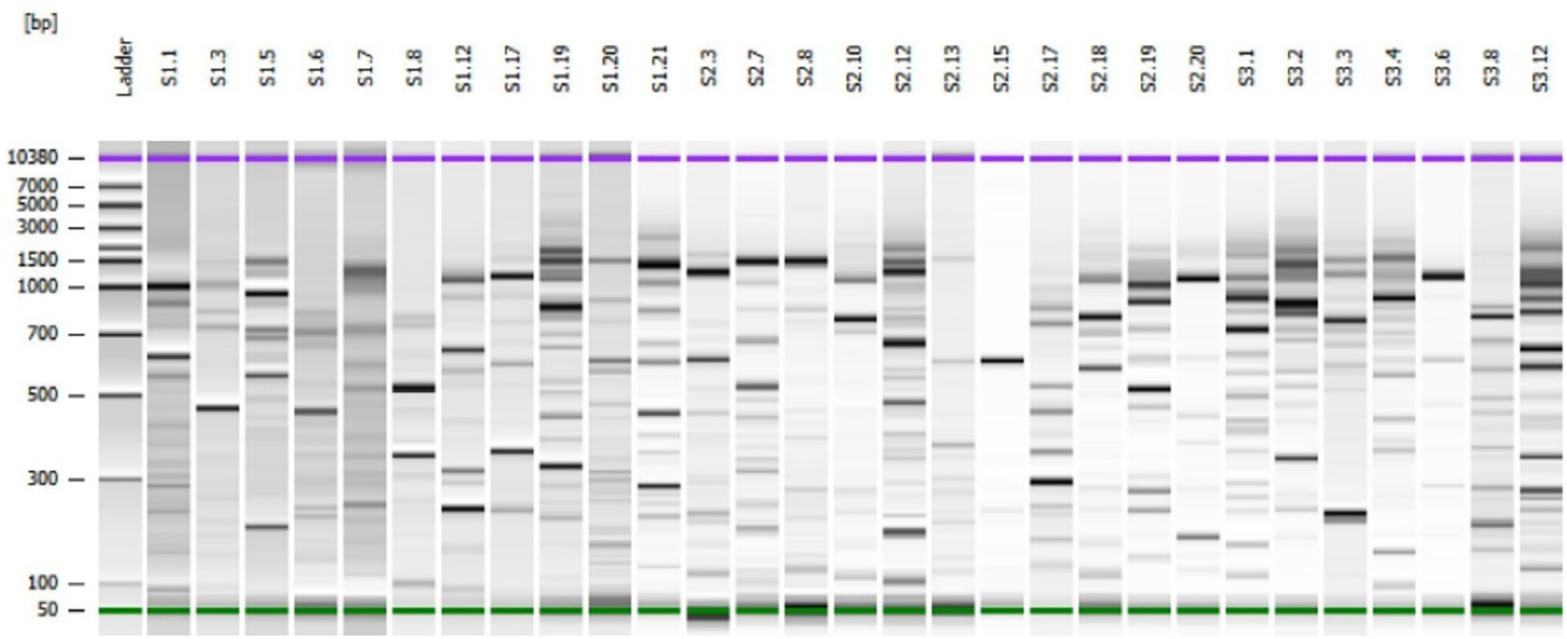
Gel-like image of rep-PCR profiles of 30 bacteria isolated from olive rhizosphere, in Tunisia.

**Figure 4:**
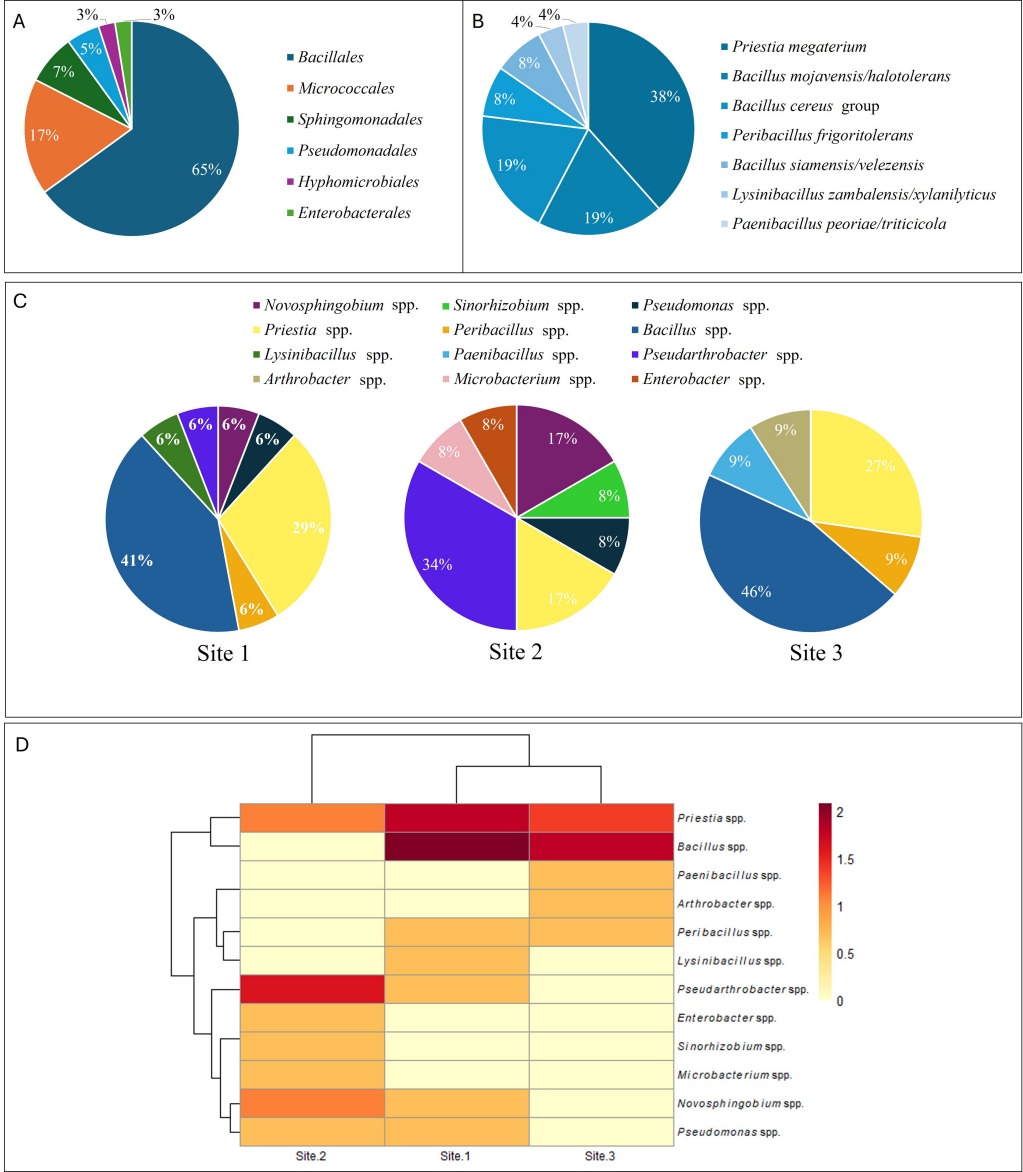
**(A)** Distribution of bacterial Order, identified by 16S rRNA gene sequencing, on olive rhizosphere, in Tunisia; **(B)** distribution of strains belonging to *Bacillales* Order. **(C)** Distribution of bacterial strains according to sampling site, site 1 (Kairouan), site 2 (Hergla, Sousse) and 3 (Sidi Bou Ali, Sousse). **(D)** Heat map of bacterial strains abundance according to sampling site. The rows represent the isolated bacterial strains, and the columns represent the sampling site. Color scale represents log-transformed abundance values.

A total of 40 bacterial strains (**Supplementary Table 2**) were isolated from the rhizosphere samples and molecularly characterized by rep-PCR to reveal clonal relationships between isolates. Results from these assays highlighted 29 different rep-PCR profiles (**Figure 3**), indicating the genetic diversity of the bacterial isolates even if obtained from the same rhizosphere soil sample and belonging to the same species. A single strain for each rep-PCR profile was identified by 16S rDNA sequencing. For each isolated bacterial strain, the species was assigned on the basis of the comparison of 16S rRNA gene sequence with the sequences of type strains deposited in NCBI database. Three strains were assigned to *Novosphingobium* genus, showing 99.4% of identity with both *N. lindaniclasticum* and *N. panipatense*. A single strain was identified as belonging to *Sinorhizobium* genus (99.85% of identity with *S. kummerowiae* and *S. meliloti* type strains). Two strains, S1C8 and S2C12, were identified as *Pseudomonas frederiksbergensis* and *Pseudomonas iranica*, respectively. Ten out of 40 bacterial strains, were identified as *Priestia megaterium*, followed by 5 strains included in the *Bacillus cereus* group, 5 strains identified as *Bacillus mojavensis*/*halotolerans* and 4 strains identified as *Pseudarthobacter siccitolerans* (**Supplementary Table 2**). At lesser extent, two *Peribacillus frigoritolerans* strains, a single *Lysinibacillus zambalensis*/*xylanilyticus* strain, two *Bacillus siamensis*/*velezensis* strains, one *Paenibacillus peoriae*/*triticicola* strain, one *Arthrobacter globiformis* strain, one *Pseudarthobacter aurescens* strain, one *Microbacterium phyllosphaerae*/f*oliorum* strain, and one *Enterobacter* spp. strain were also identified (**Supplementary Table 2**). The results showed that 65% of the isolated strains belonged to the order *Bacillales* (**Figures 4A, B**).

The distribution of these bacteria varied significantly among the sampling sites (**Figure 4C**). At Site 1 (Kairouan) and Site 3 (Sidi Bou Ali, Sousse), *Bacillus* species was overwhelmingly dominant, representing 41 and 46% of the strains, respectively which conferred them a similar profile (**Figure 4D**). In contrast, Site 2 (Hergla, Sousse) showed no presence of *Bacillus* species, and it exhibited a more balanced bacterial community, with *Pseudarthrobacter* (34%), *P. megaterium* (17%), and *Novosphingobium* (17%, **Figure 4C**).

### 3.3 Assessment of fungal pathogenicity on olive and tomato seedlings

**Figure 5:**
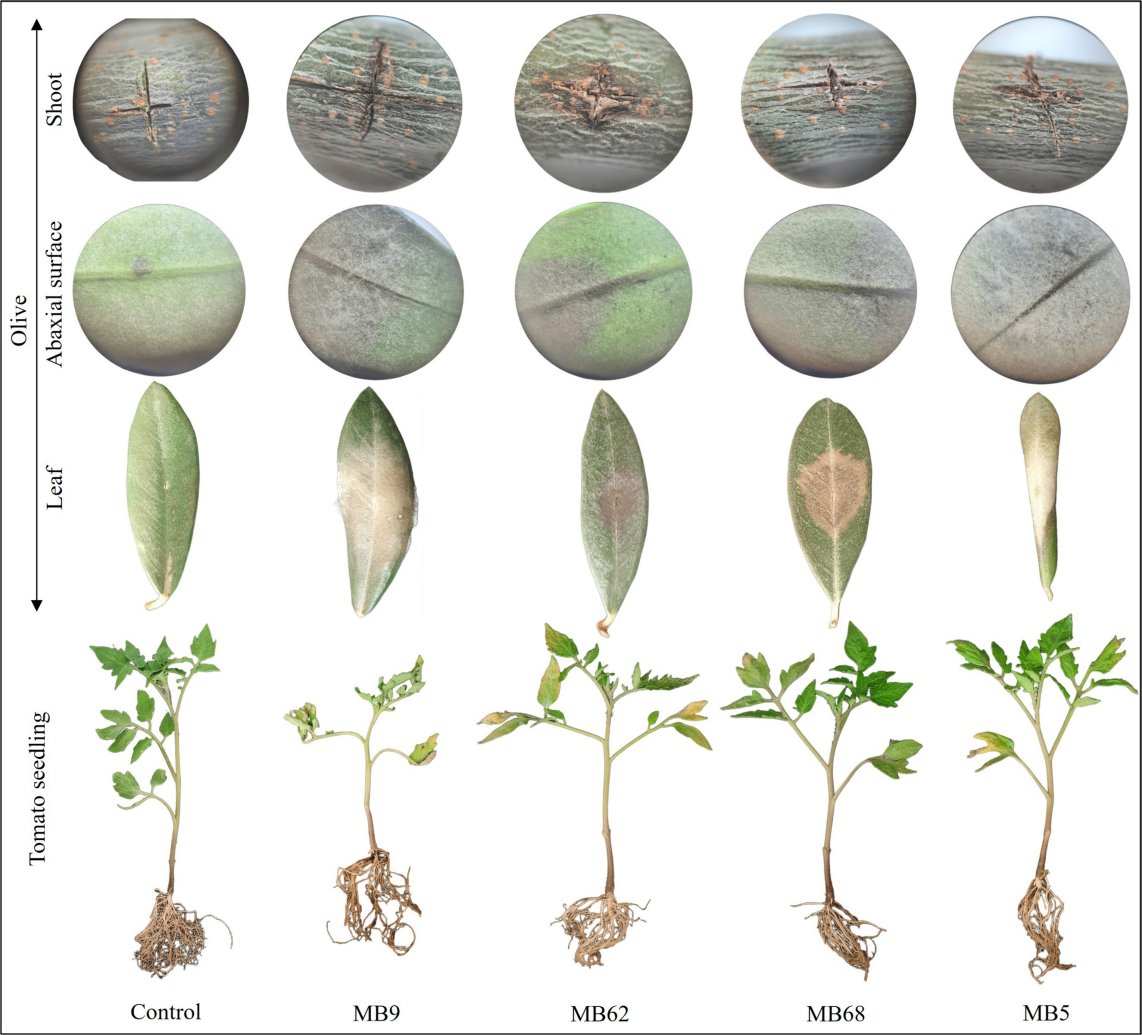
Pathogenicity test on detached olive leaves and stems and tomato seedling inoculated with strains MB9 (*Fusarium caatingaense*), MB62 (*Fusarium oxysporum*), MB68 (*Fusarium solani*) and MB5 (*Fusarium brachygibbosum*).

Seven days after inoculation, necrotic lesions developed on olive leaves (**Figure 5**; **Supplementary Figure 1**). The symptoms varied from increasing necrotic areas (MB62, MB68, MB9 and the rest of the tested strains) to the necrosis of the leaf leading to its rolling in case of some strains (*F. brachygibbosum* MB5 strain). In tomato seedlings, visible differences in vigor and root health were observed. The control plants remained healthy, with fully expanded green leaves and a well-developed root system. In contrast, seedlings inoculated with *F. caatingaense* MB9 strain showed severe stunting, chlorosis, and root deterioration, indicating strong pathogenicity. Thirty-three percent of the tested strains caused growth reduction and moderate chlorosis, while 60% had a relatively mild impact, with slight yellowing and reduced root mass to no pathogenicity effect on tomato seedlings.

The pathogenicity test on olive leaves and stems and on tomato seedlings showed that all the selected fungal strains, with the exception of the *F. nematophilum* MB57 strain and MB86 strain, belonging to *Alternaria* genus, were able to cause symptoms on olive organs and/or tomatoes seedling (**Table 1**). On olive, all tested strains were able to cause symptoms with a rate of infection ranging from 20 to 100%. On tomato seedlings, the strains MB41, MB43 MB57, MB20 and MB24 did not cause symptoms, while the other strains were pathogenic, with infection rates ranging between 25 and 100%. Members of FIESC, namely *F. longifundum*, *F. gracilipes*, and *F. caatingaense*, were pathogenic on olive (DSI values ranging between 3.57 and 28.23%) and tomato seedlings with DSI values ranging from 20 to 80%. In particular, the *F. caatingaense* MB9 strain induced the highest symptoms both on olive and tomato (**Table 1**).

**Table 1:** Disease severity index (DSI) and infection rates in tomato seedlings and olive leaves and stems, inoculated with selected fungal stains.

Strain	Fungal species	Tomato		Olive		
		DSI (%)	% Infected leaves	DSI (%)	% Infected leaves	% Infected stems
MB50	*Fusarium longifundum*	24.4 ± 5.9 cde	67	3.6 ± 1.9gh	20	50
MB56		20.9 ± 3.1 ef	50	14.8 ± 0.8e	40	50
MB72	*Fusarium gracilipes*	27.1 ± 0.4 e	67	11.8 ± 1.0ef	60	50
MB9	*Fusarium caatingaense*	80.0 ± 0.0 a	100	28.2 ± 1.0c	60	50
MB54	*Fusarium oxysporum*	11.3 ± 0.7 fg	50	23.0 ± 1.5d	100	100
MB62		51.0 ± 2.1 b	100	20.0 ± 0.7d	80	75
MB5	*Fusarium brachygibbosum*	23.0 ± 1.5 de	50	51.8 ± 1.0a	60	75
MB41		0.0 ± 0.0 h	0	8.2 ± 1.0fg	40	75
MB43		0.0 ± 0.0 h	0	45.2 ± 0.8b	40	100
MB37		5.2 ± 0.8 gh	33	5.2 ± 0.8g	0	25
MB25	*Fusarium solani*	34.0 ± 1.0 c	50	14.8 ± 0.8e	60	100
MB48		20.0 ± 2.9 ef	33	28.3 ± 0.9c	60	100
MB63		33.3 ± 2.7 cd	60	20.0 ± 0.8d	60	0
MB68		33.0 ± 2.5 cd	50	20.0 ± 1.0d	80	0
MB57	*Fusarium nemathophilum*	0.0 ± 0.0h	0	0 ± 0h	0	0
MB20	*Phoma* spp.	0.0 ± 0.0 h	0	13.4 ± 0.9e	60	0
MB59		20.3 ± 2.6 ef	25	8.3 ± 0.9fg	40	75
MB24	*Alternaria* spp.	0.0 ± 0.0 h	0	3.6 ± 1.0gh	20	50
MB86		0.0 ± 0.0h	0	0 ± 0h	0	0

Each value represents the mean ± standard error. Means with different letters within a column are significantly different at p<0.05 (Tuckey HSD test).

The four *F. brachygibbosum* strains (MB5, MB41, MB43, and MB37) showed a great diversity in causing symptoms, with DSI up to 23%, and between 5.2 and 51.8% on tomato and olive, respectively. In particular, MB5 and MB43 *F. brachygibbosum* strains were the most pathogenic on olive leaves, with DSI of 51.8 and 45.2%, respectively. All *F. solani* strains tested were pathogenic on both tomato and olive, with values ranging between 20 and 34% (**Table 1**). The two strains MB20 and MB59 belonging to *Phoma* genus, and the two strains MB24 and MB86, belonging to *Alternaria* genus, showed no or low pathogenicity on both tomato seedlings and olive leaves (**Table 1**).

### 3.4 Biocontrol potential of olive soil–derived bacteria against fungal pathogens

Twenty bacterial strains, isolated from olive rizosphere, representative of all identified bacterial species and of the different rep-PCR profiles, were tested against *F. brachygibbosum* and *F. oxysporum* species. All strains were used to evaluate their antagonistic activity by the dual culture method and six of them, selected among the most active against *Fusarium* strains were also used for studying the bacterial–fungal interactions via VOCs (**Table 2**).

**Table 2:** Activity of selected bacterial strains against *Fusarium* mycelial growth.

Bacterial strain	Bacterial species	Mycelial growth inhibition (%) of *Fusarium* strains			
		*Fusarium brachygibbosum* (MB1)		*Fusarium oxysporum* (MB54)	
		Antagonism	Indirect (VOCs)	Antagonism	Indirect (VOCs)
S1C1	*Bacillus mojavensis/halotolerans*	38.7 ± 3.3 a	38.8 ± 4.4 a	41.2 ± 2.6 b	52.6 ± 3.4 a
S1C12	*Bacillus mojavensis/halotolerans*	36.0 ± 2.5 ab	27.3 ± 5.5 ab	28.2 ± 0.0 cde	46.8 ± 1.4 a
S3C12	*Bacillus mojavensis/halotolerans*	41.2 ± 2.5 a	25.8 ± 2.7 ab	29.8 ± 3.3 bcd	29.6 ± 2.9 b
S3C1	*Bacillus siamensis/velezensis*	37.9 ± 1.1 ab	0.0 ± 0.0 c	29.79 ± 3.6 bcd	26.3 ± 2.9 bc
S1C17	*Bacillus cereus* group	29.0 ± 2.1 bc	0.0 ± 0.0 c	21.0 ± 0.8 cdef	22.4 ± 3.1 bc
S3C4	*Paenibacillus peoriae/triticicola*	29.6 ± 2.6 bc	14.3 ± 1.2 b	18.6 ± 2.0 defg	15.2 ± 0.5 c
B6	*Bacillus amyloliquefaciens*	nd	nd	67.5 ± 0.5 a	0 ± 0 d
S1C19	*Priestia megaterium*	21.5 ± 0.5 d	nd	13.6 ± 3.3 fg	nd
S1C3	*Lysinibacillus zambalensis/xylanilyticus*	19.6 ± 1.0 d	nd	31.6 ± 5.5 bc	nd
S1C5	*Peribacillus frigoritolerans*	20.6 ± 0.9 cd	nd	16.2 ± 3.2 efg	nd
S3C2	*Priestia megaterium*	37.5 ± 0.0 ab	nd	31.9 ± 2.5 bc	nd
S3C3	*Peribacillus frigoritolerans*	24.4 ± 0.6 cd	nd	8.2 ± 1.6 g	nd
S2C17	*Enterobacter* spp	26.2 ± 0.6 cd	nd	13.8 ± 1.7 fg	nd
S2C7	*Sinorhizobium kummerowiae/meliloti*	21.9 ± 1.1 cd	nd	14.1 ± 0.3 fg	nd
S1C6	*Pseudarthrobacter aurescens*	23.3 ± 0.5 cd	nd	15.7 ± 2.1 efg	nd
S2C20	*Pseudarthrobacter siccitotolerans.*	22.7 ± 1.2 cd	nd	6.1 ± 2.8 g	nd
S3C8	*Arthrobacter globiformis*	19.6 ± 1.3 d	nd	7.7 ± 0.7 g	nd
S1C8	*Pseudomonas frederiksbergensis*	37.1 ± 2.7 ab	nd	17.0 ± 1.6 efg	nd
S2C12	*Pseudomonas iranica*	22.1 ± 0.5 cd	nd	18.6 ± 0.5 defg	nd
S3C6	*Bacillus cereus* group	38.7 ± 0.6 a	nd	27.7 ± 1.2 cde	nd

Each value represents the mean ± standard error. Means with different letters within a column are significantly different at p<0.05 (Tuckey HSD test). nd, not determined

All strains inhibited mycelial growth, with variable values ranging from 19.6 (S1C3 and S3C8) to 41.2% (S3C12) and from 6.1 (S2C20) to 67.5% (B6) when tested against MB1 *F. brachygibbosum* and MB54 *F. oxysporum*, respectively (**Table 2**; **Supplementary Figure 2**).

In particular, three strains identified as *B. mojavensis*/*halotolerans* (S1C1, S1C12 and S3C12), one strain identified as *B. siamensis*/*velezensis* (S3C1) and a single *P. megaterium* strain (S3C2) were the most effective against *F. brachygibbosum*, inhibiting mycelial growth more than 35%. These strains, together with the strain S1C3 identified as *Lysinibacillus zambalensis*/*xylanilyticus* (inhibition value of 31.6%), were also effective in inhibiting mycelial growth of *F. oxysporum* (**Table 2**).

With regard to mycelial growth inhibition via VOCs, all six considered strains inhibited *F. oxysporum* mycelial growth, with values ranging between 15.2 (S3C4, *Paenibacillus peoriae*/*triticicola*) and 52.6% (S1C1, B. *mojavensis*/*halotolerans*). On the contrary, the two strains S3C1 and S1C17, showing effective antagonistic activity, did not inhibit *F. brachygibbosum* growth via VOCs (**Table 2**). Interestingly, although B6 showed the highest antagonistic activity against *F. oxysporum* strain (inhibition value of 67.5%), it had no effect via VOCs.

Since *B. amyloliquefaciens* B6 strain exhibited the highest inhibition value against *F. oxysporum*, it was further tested against other *Fusarium* strains (MB5, MB50, MB48, MB68) and MB59 strain, belonging to *Phoma* genus. To evaluate its antagonistic activity, the mycelial growth inhibition was assessed after 3 and 6 days of inoculation (**Table 3**). Fungal growth was inhibited after three days, ranging from 19.8 (MB48) to 57.5% (MB50). After six days, inhibition values increased further, reaching values between 46.3 and 74.7%, with the strongest effect recorded against *F. longifundum* MB50 strain (**Table 3**).

**Table 3:** Inhibition rate of growth (%) of MB68, MB5, MB50, MB48 and MB59 in confrontation with the *Bacillus* strain B6 at 3^rd^ and 6^th^ day after incubation (DAI) at 25°C in darkness.

Incubation period	MB50	MB5	MB59	MB68	MB48
3 DAI	57.5 ± 2.65a	53.8 ± 0.593a	48.9 ± 1.84ab	29.9 ± 0.941bc	19.8 ± 1.10c
6 DAI	74.7 ± 1.20a	68.9 ± 0.486a	69.0 ± 0.617c	50.8 ± 0.869d	46.3 ± 0.333e

Each value represents the mean ± standard error. Means within a line with different letters are significantly different at p<0.05 (Tuckey HSD test).

**Figure 6:**
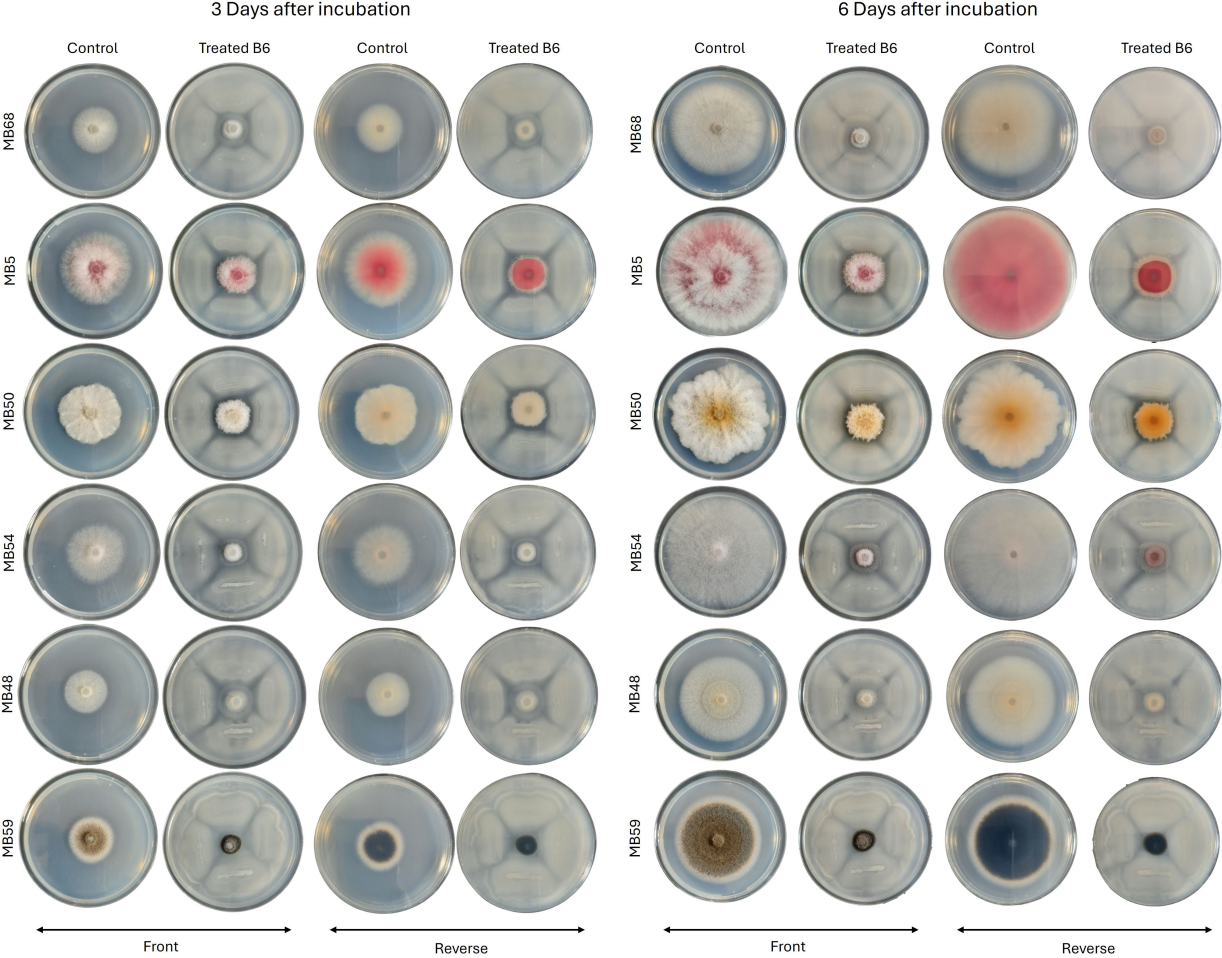
*In vitro* interaction between *Bacillus* strain B6 and MB68, MB5, MB50, MB54, MB48 and MB59 in dual culture on PDA plate at 3^rd^ and 6^th^ day after incubation at 25°C in darkness.

In this experiment, control plates exhibited normal colony development with typical morphology, whereas colonies co-cultured with B6 strain displayed markedly reduced growth (**Figure 6**). Growth inhibition was particularly evident in MB68, MB54, and MB48 strains, with mycelium appearing less dense and compact. After six days of incubation, differences between control and treated plates became even more evident. In particular, MB5, MB50, and MB54 strains cultured in presence of B6 strain remained more compact and in displayed altered pigmentation (**Figure 6**).

### 3.5 Visualization of *Bacillus* interactions with *Fusarium* and *Phoma*, using light and SEM microscopy

**Figure 7:**
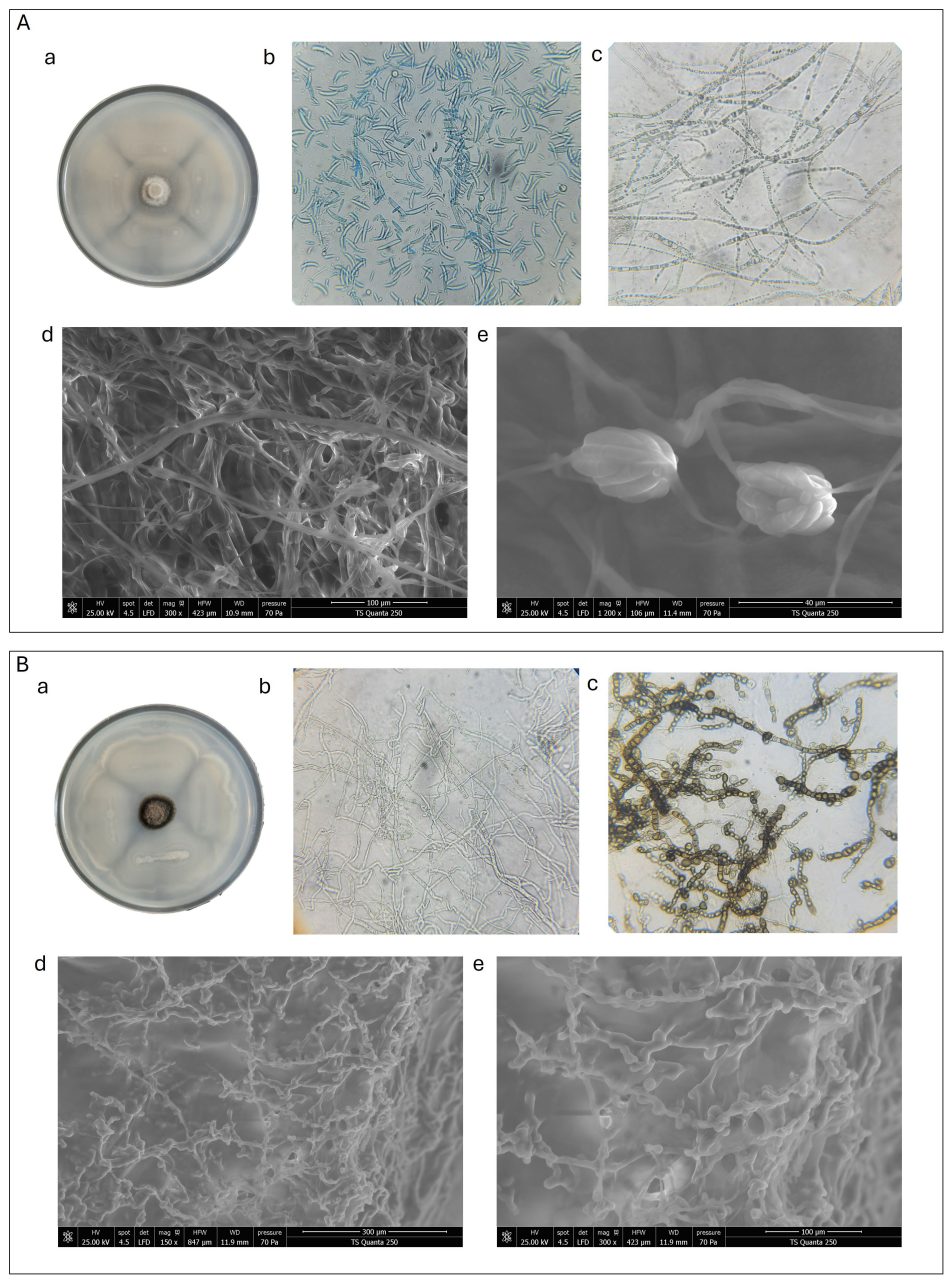
Light microscopic (x40) and SEM observations of **(A)***Fusarium solani*. Aa. fungal morphology of *Fusarium* co-culture with *Bacillus*. Ab. Light microscopic view of hyphae in control treatment without *Bacillus*. Ac, Light microscopic view of hyphae in the confrontation zone between *Fusarium* and *Bacillus*. Ad and Ae, SEM view of hyphae in the confrontation zone between *Phoma* and *Bacillus*. **(B)***Phoma* spp., Ba. fungal morphology of *Phoma* spp. co-culture with *Bacillus*. Bb. Light microscopic view of hyphae in control treatment without *Bacillus*. Bc. Light microscopic view of hyphae in the confrontation zone between *Phoma* and *Bacillus*. Bd and Be. SEM view of hyphae in the confrontation zone between *Phoma* and *Bacillus*. Images were captured on the 7th day after incubation at 25°C.

To investigate the mechanism underlying the antagonistic effect of B6 *B. amyloliquefaciens* strain, both light microscopy and SEM analyses of the interaction zone between bacterium and *F. solani* and *Phoma* strain were performed (**Figure 7**). After 7 days of co-cultivation, light microscopy revealed clear morphological changes in the hyphae and spores of both fungi compared to control. In *F. solani*, hyphae appeared irregular and distorted, with evident aggregation of conidia reflected by their reduced density (**Figures 7A–a, c**), while control samples displayed healthy, elongated hyphae with abundant, well-formed macroconidia (**Figure 7A–b**). Similarly, *Phoma* strain showed disrupted hyphal networks and dense clusters of spores (**Figures 7B–a, c**), contrasting with the more dispersed and intact hyphae in the control (**Figure 7B–b**). SEM analysis confirmed these observations, showing a dense extracellular matrix surrounding and embedding fungal hyphae and spores in the interaction zones (**Figures 7A–d, e; B–d, e**). This matrix was consistent with biofilm secretion by B6 bacterial strain, suggesting that it inhibited fungal development by producing a biofilm that physically entrapped and coagulated spores, thereby interfering with sporulation and fungal growth.

## 4 Discussion

The agricultural soils represent a reservoir of phytopathogenic microorganisms as well as of beneficial microorganisms able to play a key role in the plant-soil system. Indeed, these microorganisms can fix and transform nutrients, vital both to the plant and to other microorganisms, and have direct impact on the plant health. Microbial communities of agricultural soils can also contribute to control pathogenic microorganisms inducing systemic resistance in the plant, and producing antibiotics, siderophore compounds, and cell wall degrading enzymes (Fasusi et al., 2023; Silva et al., 2023).

The study of the microbial community colonizing the agricultural soil is of great importance to predict both their potential impact on plant health and the spread of pathogenic species. Indeed, the presence of pathogenic fungi in the soil represents not only a direct threat to the host crop but also a risk of transfer to neighboring or intercropped species, thereby amplifying disease pressure. Understanding this diversity is therefore essential to predict plant health problems and to develop sustainable management strategies.

### 4.1 Dominance of *Fusarium* in olive rhizospheres and the influence of intercropping

Molecular characterization of isolated fungal strains, based on ITS and *TEF1* gene sequencing, revealed that the fungal community associated with olive soils and roots was dominated by species belonging to *Fusarium* and *Phoma* genera. In particular, *Fusarium* was by far the most prevalent, confirming its global significance as a soilborne pathogen of numerous crops (Nikitin et al., 2023). Several *Fusarium* species, especially members of *Fusarium oxysporum* Species Complex, *Fusarium solani* Species Complex, and *Fusarium Sambucinum* Species Complex are responsible for devastating diseases such as vascular wilts and root rots, leading to substantial agricultural losses worldwide (Dean et al., 2012; Zhang et al., 2013).

In Tunisia, increasing reports of olive wilt and decline have been linked to *Fusarium* infections, although historically the focus has been on *V. dahliae* (Trabelsi et al., 2017). For decades, many *Fusarium* species were considered opportunists, mainly attacking olive trees already weakened by abiotic stress such as drought, poor soils, or insect infestation (Palmer and Kommedahl, 1969). However, the recent changes in cropping systems, including the new olive plantations on soils previously hosting *Solanaceae* plants and the expansion of olive groves intercropped with vegetables, have likely created ecological niches that favor the establishment and the spread of pathogenic *Fusarium* species. A similar trajectory has been observed for *V. dahliae*, which shifted from cotton to olive following intercropping or rotation with infected cotton plants (Jiménez-Ruiz et al., 2019; Zhang et al., 2019). These parallels highlight the role of agricultural practices and environmental factors to drive the microbial community dynamics, including the spread of soilborne fungi.

In 2017, Trabelsi et al. reported a high presence of *Fusarium* species, including *F. solani* and *F. oxysporum*, in Tunisian olive groves, where 81 out of 104 *Fusarium* strains were found to be nonpathogenic. In contrast, the present work provides evidence that links the emergence of pathogenic *Fusarium* populations to intercropping practices, suggesting that cropping systems may act as a key driver in the transition of these species from minor microbiome components to significant olive pathogens. Pathogenicity was assessed using detached olive leaves and stems, while root infection assays were not conducted due to experimental limitations. As *Fusarium* species are primarily soil-borne pathogens, symptoms observed on detached tissues may not fully represent the systemic nature of *Fusarium* as a vascular pathogen. Previous studies have shown that *Fusarium* culture filtrates and purified mycotoxins can induce necrosis and chlorosis on detached plant tissues independently of fungal colonization, highlighting the ability of *Fusarium* secondary metabolites to cause tissue damage *in vitro* (Perincherry et al., 2019; Xu et al., 2021). Therefore, the necrotic symptoms observed in this study may reflect not only the ability of these *Fusarium* strains to colonize olive plant tissues but also their potential toxigenicity.

*Fusarium brachygibbosum* represented nearly one-third of all *Fusarium* isolates in our samples. Although previously reported in olive by (Trabelsi et al., 2017), its relatively high frequency and detection across all sites highlight its emerging importance in Tunisian olive groves and its role in olive wilt decline. Given its wide host range and recent designation as a potential quarantine pest in the European Union (EFSA Panel on Plant et al., 2021), this species deserves particular attention. In addition, members of FSSC were the second most represented group (≈31%) in agreement with earlier findings (Trabelsi et al., 2017).

Meanwhile, members of FIESC accounted for approximately 22% of the isolated *Fusarium* species. Species of this complex are increasingly associated with tree hosts, including Aleppo Pine in Algeria (Lazreg et al., 2014), *Eucommia ulmoides* in China (Fang et al., 2020), citrus tree in Lebanon (Abi Saad et al., 2022) and date-palms in the south of Tunisia (Rabaaoui et al., 2021), suggesting that they produce enzymatic repertoires adapted for colonizing lignified tissues. Notably, infections by multiple *Fusarium* species have been shown to exacerbate disease severity compared to single-species infections (Zhang et al., 2022), which may explain the severity of olive decline observed in some groves. By contrast, *F. nematophilum* was rarely isolated, and consistent with recent reports, appears to act more as a beneficial endophyte or growth promoter rather than as pathogen (Yan et al., 2024, 2025).

Other fungal genera, although less abundant, may still influence olive health under certain conditions. *Alternaria* and *Phoma* include both pathogenic and saprophytic species. *Alternaria* species are known for producing phytotoxins and have been implicated in leaf spot diseases of olive (Basım et al., 2017; Tziros et al., 2021). However, they are frequently isolated from stressed or *Xylella*-infected olive trees, suggesting opportunistic colonization (Giampetruzzi et al., 2020; Lamendola et al., 2024). *Phoma* species have been associated to branch dieback in Tunisian olives (Rhouma et al., 2010), often in conjunction with environmental stressors. Ubiquitous genera such as *Aspergillus* and *Penicillium* were also recovered, reflecting the complex microbial community of olive groves (Gharsallah et al., 2023).

Altogether, these findings highlight the dominance of *Fusarium* in Tunisian olive rhizospheres and underscore the role of intercropping practices in shaping microbial communities. Although plants intercropping can introduce beneficial diversity, it may also favor the spread of soil-borne pathogens, posing risks for both olive and associated crops. The distribution of *Fusarium* species varied markedly among the surveyed sites, reflecting the influence of intercropping and site-specific management. In Site 1 (Kairouan), where olives were intercropped with potato for seven crop seasons, *Fusarium* accounted for only 25% of isolates, while *Alternaria* and other fungi dominated. In contrast, Sites 2 and 3 (Hergla and Sidi Bou Ali), with less diverse and long-term intercropping systems with *Solanaceae* plants, showed *Fusarium* as the predominant genus, reaching 54% and 80% of the strains, respectively. More generally, intercropping systems that increase host continuity or include highly susceptible crops may promote the persistence and accumulation of soil-borne fungi, whereas diversified crop rotations can disrupt pathogen life cycles and reduce overall fungal dominance (Huss et al., 2022). This was particularly observed in Site 3, where *Fusarium* species were the most dominant in the isolated fungal strains, likely due to growing tomato crops between olive rows, which is considered a susceptible host to *Fusarium*. Similar findings have been reported in other cropping systems where plant diversification was associated with lower incidence of soil-borne pathogens, likely due to changes in root exudate composition, increased microbial antagonism, and improved soil structure. In olives, intercropping with annual crops has been shown to influence both soil nutrient dynamics and microbial communities, with potential consequences for disease pressure (Trabelsi et al., 2017). Additionally, the absence of *Verticillium* strains among the isolated strains was notable. Similar findings were previously reported where typical *Verticillium* wilt symptoms were observed but the pathogen was not consistently re-isolated from symptomatic plants (ElDesouki-Arafat et al., 2021; Serrano et al., 2021). These studies emphasize the slow growth of *Verticillium* on general media, allowing faster-growing fungal taxa to dominate.

### 4.2 Dominance and distribution of bacterial species in olive rhizospheres

The molecular identification of bacteria isolated from olive rhizosphere samples revealed that the bacterial community was largely composed by *Firmicutes*, *Proteobacteria*, and *Actinobacteria*, consistent with previous studies in olive trees (Dias et al., 2024; Gharsallah et al., 2023; Melloni and Cardoso, 2023). Among the 40 isolated strains, *Bacillus* was the most frequently identified genus, as observed in previous studies carried out in different arid areas of Tunisia, including Sfax region (Gharsallah et al., 2023). The high presence of *Bacillus* species confirms the great adaptability of these bacteria to environmental conditions, including the tolerance to desiccation and UV radiation (Gharsallah et al., 2023).

Site-specific variations were observed, since in Site 2, *Bacillus* was not detected, probably due to specific soil attributes, as previously suggested for other olive orchards (Thenappan et al., 2024). Only one *P. megaterium* strain was recovered from this site, supporting the influence of local environmental factors on microbial recovery. Interestingly, despite *Proteobacteria* typically being dominant in olive root microbiomes (Giampetruzzi et al., 2020; Vergine et al., 2020), no *Proteobacteria* were isolated from Site 3, further illustrating site-specific variability. The presence of *Enterobacter* in this site may reflect shifts in agronomic practices, such as increased irrigation and potential use of lower-quality water sources (Baudišová et al., 2022).

The distribution patterns of bacterial and fungal communities across the three sites suggest possible ecological interactions that may influence pathogen dynamics in olive orchards. Sites 2 and 3, where *Fusarium* was overwhelmingly dominant, showed different bacterial species profiles. In Site 2, *Bacillus* was absent and only one *P. megaterium* strain was recovered, while *Proteobacteria* were present. In Site 3, *Bacillus* was dominant, but no *Proteobacteria* were isolated. Conversely, in intercropped Site 1, *Fusarium* abundance was lower and *Bacillus* co-occurred with other bacterial taxa, suggesting that bacterial diversity may contribute to suppressing fungal dominance. Disease suppressiveness is recognized as an emergent property of complex microbial community interactions (Todorović et al., 2023). Previous studies also indicate that although *Bacillus* spp. can effectively inhibit *Fusarium* through the production of antifungal metabolites and competitive interactions under controlled conditions (Yang et al., 2024), such antagonistic effects are highly strain-dependent and strongly influenced by environmental factors. Therefore, simple abundance patterns do not necessarily reflect functional antagonism *in situ* (Sun et al., 2022).

These observations align with the well-documented antagonistic potential of *Firmicutes*, particularly *Bacillus* species, against soilborne pathogens such as *Fusarium* (Han et al., 2019). *Bacillus* can inhibit fungal growth through multiple mechanisms, including production of antibiotics, volatile organic compounds, and biofilm formation, creating physical and chemical barriers to pathogen establishment. Therefore, the co-occurrence of diverse bacterial communities in Site 1 may partially explain the reduced dominance of *Fusarium*, whereas the absence or low abundance of antagonistic bacteria in Sites 2 could have facilitated pathogen proliferation and the observed emergence of pathogenic strains.

These findings suggest a complex interplay between bacterial and fungal communities, where site-specific environmental conditions and cropping practices modulate microbial interactions, ultimately influencing pathogen dynamics and disease risk in olive groves. This highlights the importance of integrating microbial community management, including promotion of beneficial bacteria, as a strategy for mitigating soil-borne pathogen threats.

### 4.3 Native bacterial antagonists as biocontrol agents in olive orchards

The bacterial strains recovered from olive soils exhibited variable *in vitro* antagonistic activity against *Fusarium* and *Phoma* species, highlighting the potential of soil-derived microbes as biocontrol agents. In dual culture assays, *B. amyloliquefaciens* B6 strain displayed the strongest inhibition activity, followed by other *Bacillus* isolates showing moderate inhibition activity. Other genera, including *Bacillus cereus* group, *Paenibacillus*, *Priestia*, *Peribacillus*, *Pseudarthrobacter*, *Enterobacter*, *Sinorhizobium*, and *Pseudomonas*, exhibited intermediate to low inhibition. These results indicate that the genus *Bacillus* is particularly effective in suppressing fungal growth, in line with previous reports of its broad-spectrum antifungal properties (Chen et al., 2009).

Interestingly, VOCs assays revealed that the mechanisms of antagonism varied among strains and species. Although B6 showed the highest direct inhibition in dual culture, it exhibited no effect via VOCs. In contrast, *Bacillus* strains S1C1 and S1C12 demonstrated strong VOCs-mediated inhibition, suggesting that different strains employ distinct antifungal strategies, including production of VOCs that can inhibit fungal growth (Raaijmakers et al., 2010; Sharifi and Ryu, 2018).

Further temporal analysis of B6 against highly pathogenic *Fusarium* strains showed that inhibition was evident as early as 3 days after inoculation and increased by 6 days up to 74.7%. The rapid onset of growth suppression highlights the strong and consistent antagonistic potential of B6, capable of acting quickly to limit pathogen development. Colonies exposed to B6 exhibited reduced hyphal density, compact morphology, and altered pigmentation, reflecting physiological stress and growth inhibition. These observations are based on *in vitro* assays and require further validation *in planta* to confirm their relevance under whole-plant conditions.

Light microscopy and SEM further elucidated the mechanism of antagonism. At the interaction zones, hyphae of *F. solani* and *Phoma* appeared distorted and aggregated, and spores clustered densely compared to controls. SEM images revealed a dense extracellular matrix embedding fungal hyphae and spores, consistent with biofilm production by B6. This suggests a biofilm-mediated mechanism in which the bacterium physically traps and coagulates fungal structures, interfering with sporulation and mycelial expansion. Such biofilm-mediated inhibition has been reported for other *Bacillus* strains, providing both physical and chemical barriers against phytopathogens (Bais et al., 2004; Ongena and Jacques, 2008).

Overall, these findings demonstrate that *Bacillus* species activate multiple strategies to interact with other microorganisms including antagonism, direct mycelial inhibition, VOCs production, and biofilm-mediated entrapment. Their activity is likely influenced by both strain-specific traits and environmental conditions, highlighting the importance of selecting robust native isolates for biocontrol applications. The integration of such bacterial biocontrol agents in olive and vegetable cropping systems could reduce *Fusarium* and *Phoma* incidence, complementing cultural practices such as intercropping and promoting sustainable disease management in Mediterranean orchards (Raaijmakers et al., 2010).

## 5 Conclusion

This study provides a comprehensive overview of the microbial communities in Tunisian olive soils and roots, highlighting interactions between fungi and bacteria and the influence of cropping practices. *Fusarium* was the dominant fungal genus, with its abundance varying across sites. The highest prevalence and severity were observed in Sidi Bou Ali, where the presence of tomato a highly susceptible crop likely contributed to the proliferation of virulent *Fusarium* strains. Notably, compared to earlier reports, the pathogenicity of *Fusarium* in these olive orchards has increased over the past seven years, indicating a rise in disease risk. The *in vitro* assay showed that bacterial communities, particularly *Bacillus* species, exhibited strong antagonistic potential against *Fusarium* and *Phoma*, employing diverse mechanisms including direct mycelial inhibition, VOCs, and biofilm-mediated entrapment which should further be validated through *in planta* assays. Overall, these findings emphasize the importance of considering site-specific cropping systems, host crop susceptibility, and native bacterial communities in managing soilborne pathogens. Leveraging indigenous biocontrol bacteria alongside informed crop management offers a promising strategy to reduce disease risk and support sustainable olive cultivation in Mediterranean agroecosystems.

## Data Availability

The original contributions presented in the study are publicly available. This data can be found here: NCBI Nucleotide database, https://www.ncbi.nlm.nih.gov/nuccore/, accessions: 16S rDNA, PZ098755–PZ098783; ITS rDNA, PZ111392–PZ111468; TEF-1α, PZ121336–PZ121387.
