# Supplementary material for: Microbial biodiversity in Tunisian olive grove soils: a reservoir of phytopathogenic fungi and potential beneficial microorganisms
Source: Front Fungal Biol. 2026 Mar 16;7:1770745. doi: 10.3389/ffunb.2026.1770745 (PMC13033644; doi:10.3389/ffunb.2026.1770745)
Supplement: Supplementary file 1 [file Supplementaryfile1.docx]

Supplementary Material

# Supplementary figures


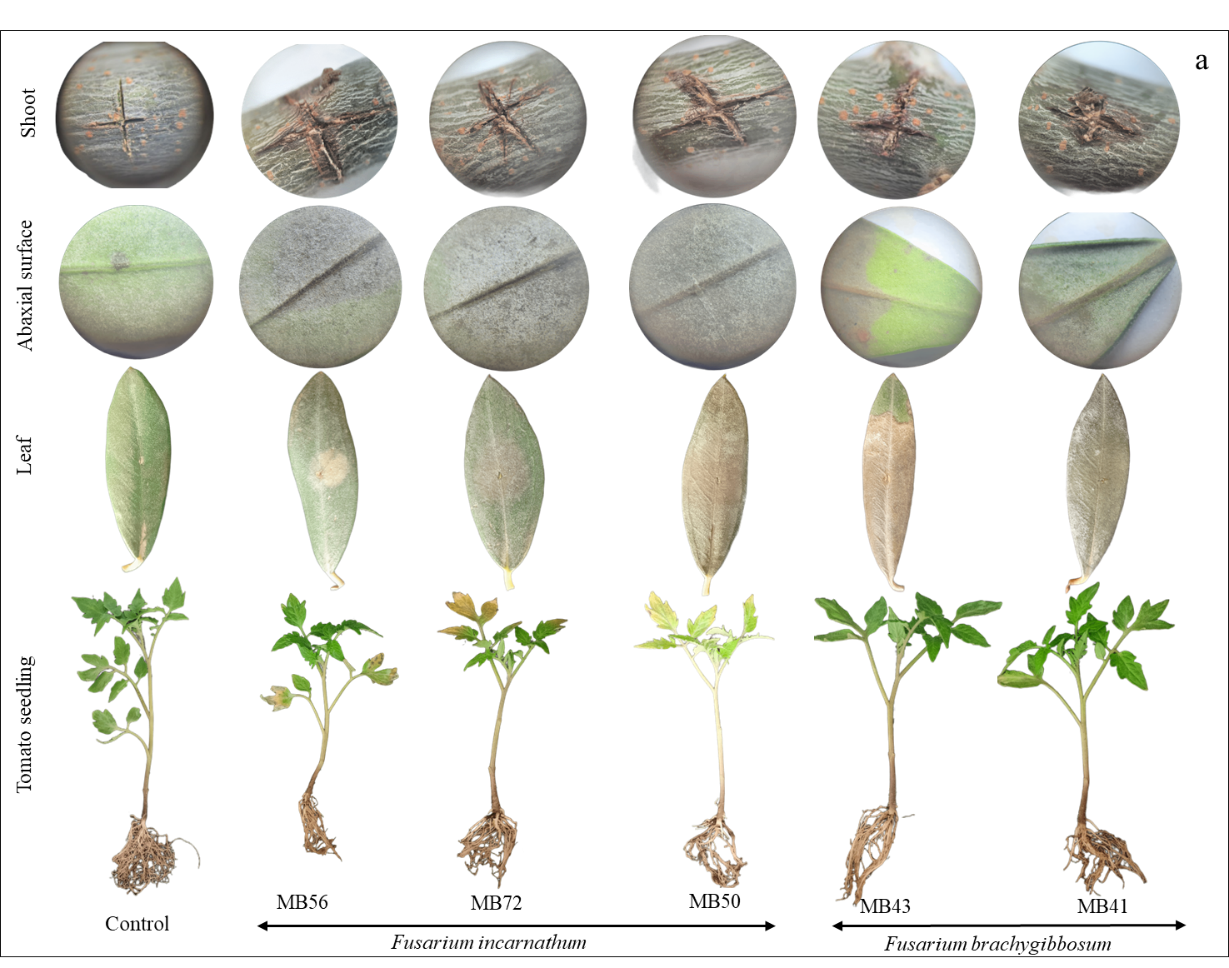


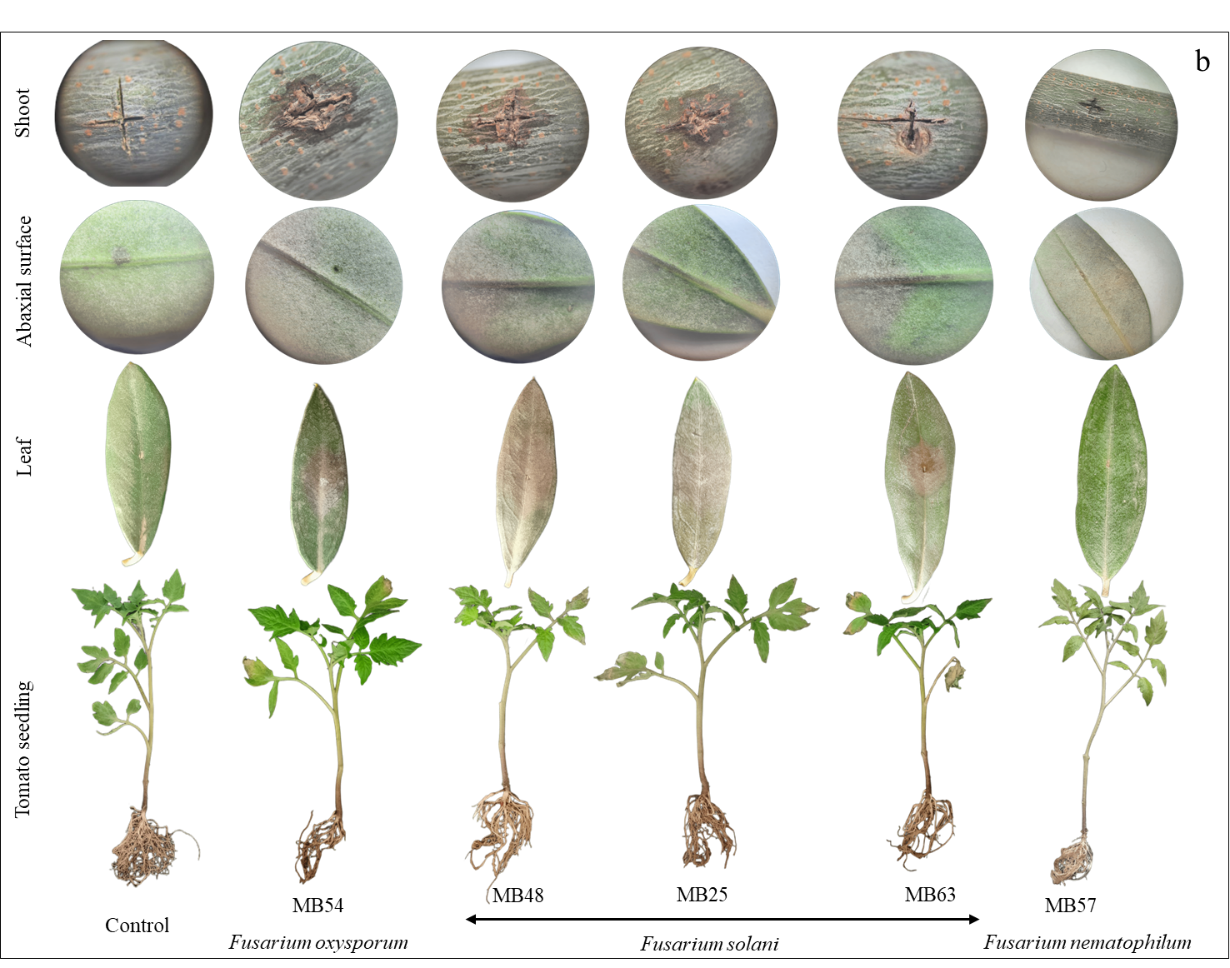


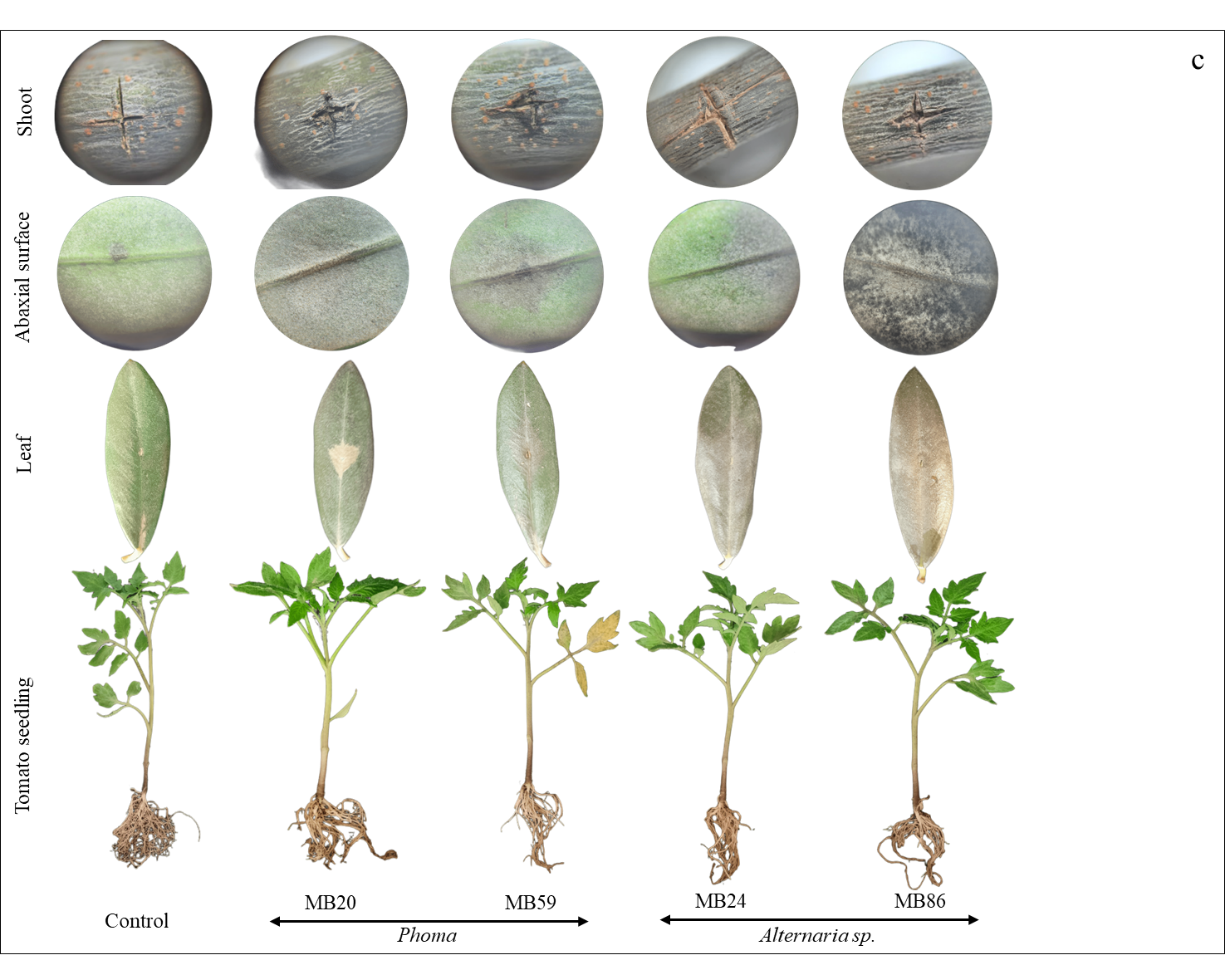


**Supplementary Figure 1.** Pathogenicity test of (a and b) *Fusarium* sp. and (c) *Phoma* and *Alternaria* sp. on olive leaves (adaxial and abaxial surfaces), olive shoots, and tomato seedlings. Observations of the abaxial surface of olive leaves and olive shoots were conducted using a binocular magnifier.


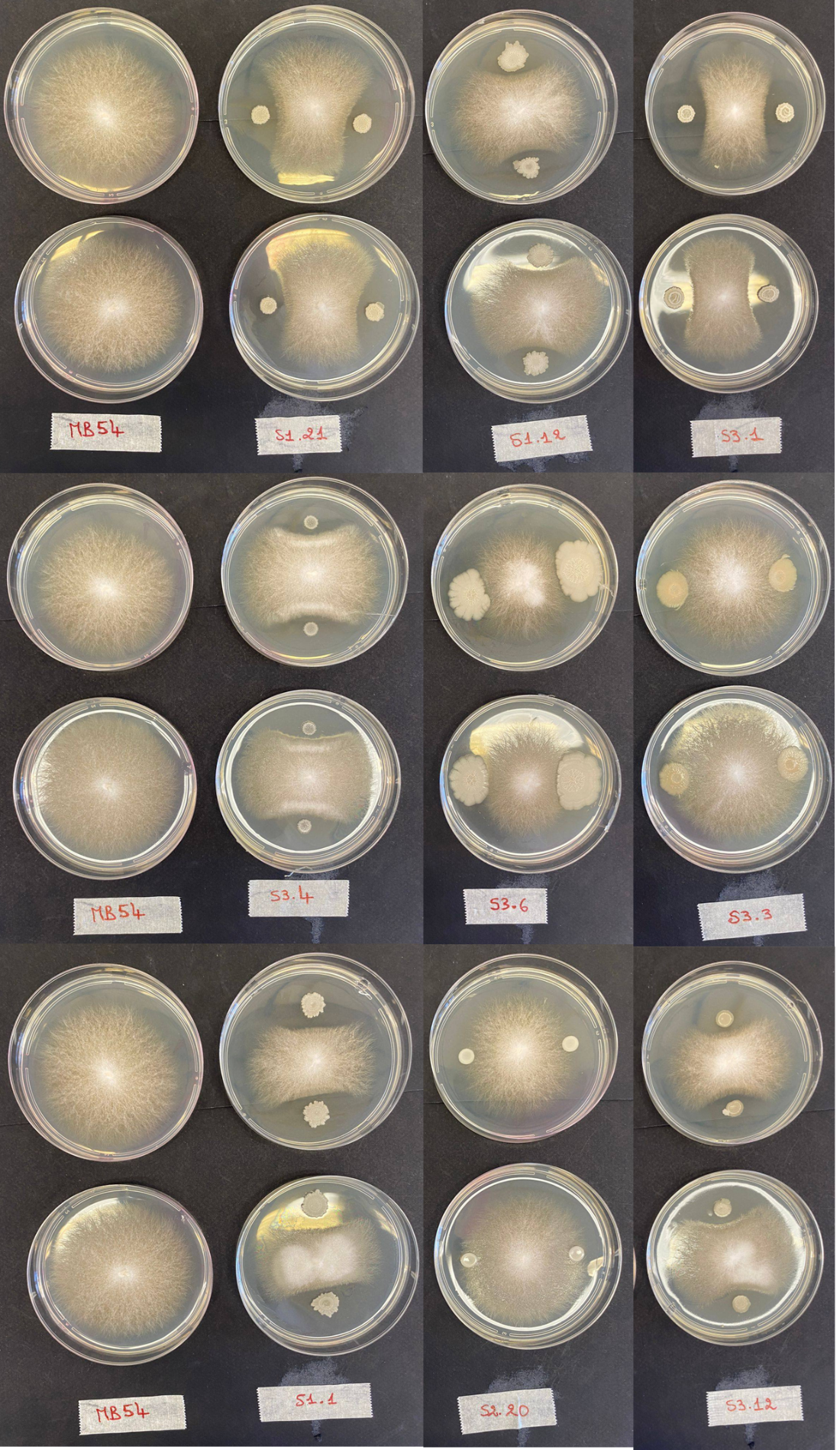


**Supplementary Figure 2.** In vitro interaction between bacterial strains and MB54 in dual culture on PDA plate at 7^th^ day after incubation at 25°C in 12h photoperiod.

# Supplementary tables

**Supplementary Table 1.** Fungal strains isolated from olive groves in Tunisia, used in this study.

| **Fungal species** | **Sampling site** | **Origin** | **Strain** | **ITS Accession Number** | **TEF Accession number** |
| --- | --- | --- | --- | --- | --- |
| *Fusarium brachygibbosum* | Site 1 | roots | MB1 | - | PZ121336 |
| *Fusarium brachygibbosum* | Site 1 | roots | MB2 | - | PZ121337 |
| *Fusarium brachygibbosum* | Site 2 | roots | MB5 | - | PZ121338 |
| *Fusarium brachygibbosum* | Site 2 | roots | MB6 | PZ111392 | PZ121339 |
| *Fusarium brachygibbosum* | Site 2 | roots | MB8 | PZ111393 | PZ121340 |
| *Fusarium brachygibbosum* | Site 3 | roots | MB36 | PZ111394 | PZ121352 |
| *Fusarium brachygibbosum* | Site 3 | roots | MB37 | PZ111395 | PZ121353 |
| *Fusarium brachygibbosum* | Site 3 | roots | MB39 | PZ111396 | PZ121355 |
| *Fusarium brachygibbosum* | Site 3 | roots | MB40 | PZ111397 | PZ121356 |
| *Fusarium brachygibbosum* | Site 3 | roots | MB41 | PZ111398 | PZ121357 |
| *Fusarium brachygibbosum* | Site 3 | roots | MB43 | PZ111399 | PZ121359 |
| *Fusarium brachygibbosum* | Site 3 | rhizosphere | MB53 | PZ111400 | PZ121366 |
| *Fusarium brachygibbosum* | Site 2 | roots | MB67 | PZ111401 | PZ121375 |
| *Fusarium brachygibbosum* | Site 2 | roots | MB69 | PZ111402 | PZ121377 |
| *Fusarium brachygibbosum* | Site 3 | roots | MB75 | PZ111403 | PZ121381 |
| *Fusarium brachygibbosum* | Site 3 | roots | MB77 | PZ111404 | PZ121382 |
| *Fusarium brachygibbosum* | Site 3 | roots | MB78 | PZ111405 | PZ121383 |
| *Fusarium caatingaense* | Site 2 | roots | MB9 | PZ111406 | PZ121341 |
| *Fusarium clavum* | Site 3 | rhizosphere | MB52 | PZ111407 | PZ121365 |
| *Fusarium gracilipes* | Site 3 | roots | MB72 | PZ111408 | PZ121380 |
| *Fusarium incarnatum* | Site 2 | roots | MB10 | - | PZ121342 |
| *Fusarium longifundum* | Site 2 | roots | MB12 | - | PZ121343 |
| *Fusarium longifundum* | Site 3 | roots | MB35 | PZ111409 | PZ121351 |
| *Fusarium longifundum* | Site 3 | roots | MB42 | PZ111410 | PZ121358 |
| *Fusarium longifundum* | Site 3 | roots | MB47 | PZ111411 | PZ121361 |
| *Fusarium longifundum* | Site 3 | roots | MB50 | PZ111412 | PZ121364 |
| *Fusarium longifundum* | Site 3 | roots | MB56 | PZ111413 | PZ121369 |
| *Fusarium longifundum* | Site 3 | roots | MB70 | PZ111414 | PZ121378 |
| *Fusarium nematophilum* | Site 2 | roots | MB30 | PZ111415 | PZ121350 |
| *Fusarium nematophilum* | Site 3 | roots | MB57 | PZ111416 | PZ121370 |
| *Fusarium nematophilum* | Site 2 | roots | MB61 | PZ111417 | PZ121371 |
| *Fusarium nematophilum* | Site 2 | roots | MB65 | - | PZ121374 |
| *Fusarium oxysporum* | Site 3 | roots | MB49 | PZ111418 | PZ121363 |
| *Fusarium oxysporum* | Site 3 | roots | MB54 | PZ111419 | PZ121367 |
| *Fusarium oxysporum* | Site 2 | roots | MB62 | PZ111420 | PZ121372 |
| *Fusarium solani* | Site 2 | roots | MB16 | PZ111421 | PZ121344 |
| *Fusarium solani* | Site 2 | roots | MB63 | PZ111422 | PZ121373 |
| *Fusarium solani* | Site 3 | roots | MB71 | PZ111423 | PZ121379 |
| *Fusarium solani* | Site 2 | roots | MB17 | PZ111424 | PZ121345 |
| *Fusarium solani* | Site 2 | roots | MB18 | PZ111425 | PZ121346 |
| *Fusarium solani* | Site 2 | roots | MB19 | PZ111426 | PZ121347 |
| *Fusarium solani* | Site 2 | roots | MB25 | PZ111427 | PZ121348 |
| *Fusarium solani* | Site 2 | roots | MB26 | PZ111428 | PZ121349 |
| *Fusarium solani* | Site 3 | roots | MB45 | PZ111429 | PZ121360 |
| *Fusarium solani* | Site 3 | roots | MB48 | PZ111430 | PZ121362 |
| *Fusarium solani* | Site 3 | roots | MB55 | PZ111431 | PZ121368 |
| *Fusarium solani* | Site 2 | roots | MB68 | PZ111432 | PZ121376 |
| *Fusarium solani* | Site 3 | roots | MB79 | PZ111433 | PZ121384 |
| *Fusarium solani* | Site 3 | roots | MB80 | PZ111434 | PZ121385 |
| *Fusarium solani* | Site 3 | roots | MB82 | PZ111435 | PZ121386 |
| *Fusarium solani* | Site 3 | roots | MB83 | PZ111436 | PZ121387 |
| *Alternaria* spp. | Site 1 | rhizosphere | MB4 | PZ111437 | - |
| *Alternaria* spp. | Site 2 | roots | MB21 | PZ111438 | - |
| *Alternaria* spp. | Site 2 | rhizosphere | MB24 | PZ111439 | - |
| *Alternaria* spp. | Site 3 | roots | MB76 | PZ111440 | - |
| *Alternaria* spp. | Site 1 | roots | MB85 | PZ111441 | - |
| *Alternaria* spp. | Site 1 | roots | MB86 | PZ111442 | - |
| *Alternaria* spp. | Site 1 | roots | MB87 | PZ111443 | - |
| *Alternaria* spp. | Site 1 | roots | MB88 | PZ111444 | - |
| *Aspergillus* spp. | Site 2 | rhizosphere | MB27 | PZ111445 | - |
| *Aspergillus* spp. | Site 2 | roots | MB29 | PZ111446 | - |
| *Aspergillus* spp. | Site 2 | roots | MB33 | PZ111447 | - |
| *Aspergillus* spp. | Site 2 | roots | MB66 | PZ111448 | - |
| *Aspergillus* spp. | Site 3 | roots | MB73 | PZ111449 | - |
| *Chaetomium* spp. | Site 2 | rhizosphere | MB34 | PZ111450 | - |
| *Chaetomium* spp. | Site 2 | roots | MB60 | PZ111451 | - |
| *Clonostachys rosea* | Site 3 | roots | MB38 | PZ111452 | PZ121354 |
| *Exophiala* spp. | Site 1 | roots | MB84 | PZ111453 | - |
| *Mortierella* spp. | Site 1 | rhizosphere | MB3 | PZ111454 | - |
| *Mortierella* spp. | Site 2 | rhizosphere | MB28 | PZ111455 | - |
| *Phoma* spp. | Site 2 | roots | MB15 | PZ111456 | - |
| *Phoma* spp. | Site 2 | rhizosphere | MB20 | PZ111457 | - |
| *Phoma* spp. | Site 2 | roots | MB22 | PZ111458 | - |
| *Phoma* spp. | Site 2 | roots | MB23 | PZ111459 | - |
| *Phoma* spp. | Site 3 | rhizosphere | MB44 | PZ111460 | - |
| *Phoma* spp. | Site 3 | roots | MB46 | PZ111461 | - |
| *Phoma* spp. | Site 3 | roots | MB51 | PZ111462 | - |
| *Phoma* spp. | Site 2 | roots | MB58 | PZ111463 | - |
| *Phoma* spp. | Site 2 | roots | MB59 | PZ111464 | - |
| *Phoma* spp. | Site 2 | roots | MB64 | PZ111465 | - |
| *Phoma* spp. | Site 1 | roots | MB90 | PZ111466 | - |
| *Thelonectria* spp. | Site 2 | roots | MB32 | PZ111467 | - |
| *Thelonectria* spp. | Site 3 | roots | MB74 | PZ111468 | - |

**Supplementary Table 2.** Bacterial strains isolated from olive rhizosphere in Tunisia, used in this study.

| **Bacterial species** | **Sampling site** | **Origin** | **% of 16S rRNA** | **Strain** | **Accession Number** |
| --- | --- | --- | --- | --- | --- |
|  |  |  | **gene identity** |  |  |
| *Bacillus cereus* group | Site 1 | rhizosphere | - | S1C4 | - |
| *Bacillus cereus* group | Site 1 | rhizosphere | - | S1C17 | PZ098767 |
| *Bacillus cereus* group | Site 3 | rhizosphere | - | S3C6 | PZ098768 |
| *Bacillus cereus* group | Site 3 | rhizosphere | - | S3C11 | - |
| *Bacillus cereus* group | Site 3 | rhizosphere | - | S3C13 | - |
| *Bacillus mojavensis/halotolerans* | Site 1 | rhizosphere | 99.93 | S1C1 | PZ098762 |
| *Bacillus mojavensis/halotolerans* | Site 1 | rhizosphere | 99.93 | S1C7 | PZ098760 |
| *Bacillus mojavensis/halotolerans* | Site 1 | rhizosphere | 99.93 | S1C9 | - |
| *Bacillus mojavensis/halotolerans* | Site 1 | rhizosphere | 99.93 | S1C12 | PZ098763 |
| *Bacillus mojavensis/halotolerans* | Site 3 | rhizosphere | 99.93 | S3C12 | PZ098761 |
| *Bacillus siamensis/velezensis* | Site 1 | rhizosphere | 100 | S1C21 | PZ098764 |
| *Bacillus siamensis/velezensis* | Site 3 | rhizosphere | 100 | S3C1 | PZ098765 |
| *Arthrobacter globiformis* | Site 3 | rhizosphere | 99.47 | S3C8 | PZ098774 |
| *Enterobacter* spp. | Site 2 | rhizosphere | - | S2C17 | PZ098782 |
| *Lysinibacillus zambalensis/xylanilyticus* | Site 1 | rhizosphere | 99.93 | S1C3 | PZ098766 |
| *Microbacterium phyllosphaerae/foliorum* | Site 2 | rhizosphere | 99.85 | S2C15 | PZ098776 |
| *Novosphingobium lindaniclasticum* | Site 2 | rhizosphere | 99.4 | S2C10 | PZ098778 |
| *Novosphingobium lindaniclasticum* | Site 2 | rhizosphere | 99.4 | S2C18 | PZ098783 |
| *Novosphingobium panipatense* | Site 1 | rhizosphere | 99.4 | S1C20 | PZ098777 |
| *Paenibacillus peoriae/triticicola* | Site 3 | rhizosphere | 99.93 | S3C4 | PZ098769 |
| *Peribacillus frigoritolerans* | Site 1 | rhizosphere | 99.93 | S1C5 | PZ098755 |
| *Peribacillus frigoritolerans* | Site 3 | rhizosphere | 99.93 | S3C3 | PZ098756 |
| *Priestia megaterium* | Site 1 | rhizosphere | 100 | S1C2 | - |
| *Priestia megaterium* | Site 1 | rhizosphere | 100 | S1C11 | - |
| *Priestia megaterium* | Site 1 | rhizosphere | 100 | S1C14 | - |
| *Priestia megaterium* | Site 1 | rhizosphere | 100 | S1C15 | - |
| *Priestia megaterium* | Site 1 | rhizosphere | 100 | S1C19 | PZ098757 |
| *Priestia megaterium* | Site 2 | rhizosphere | 100 | S2C16 | - |
| *Priestia megaterium* | Site 2 | rhizosphere | 100 | S2C19 | PZ098758 |
| *Priestia megaterium* | Site 3 | rhizosphere | 100 | S3C2 | PZ098759 |
| *Priestia megaterium* | Site 3 | rhizosphere | 100 | S3C9 | - |
| *Priestia megaterium* | Site 3 | rhizosphere | 100 | S3C10 | - |
| *Pseudarthrobacter aurescens* | Site 1 | rhizosphere | 99.93 | S1C6 | PZ098775 |
| *Pseudarthrobacter siccitolerans* | Site 2 | rhizosphere | 99.2 | S2C3 | PZ098772 |
| *Pseudarthrobacter siccitolerans* | Site 2 | rhizosphere | 99.2 | S2C8 | PZ098773 |
| *Pseudarthrobacter siccitolerans* | Site 2 | rhizosphere | 99.2 | S2C11 | PZ098770 |
| *Pseudarthrobacter siccitolerans* | Site 2 | rhizosphere | 99.2 | S2C20 | PZ098771 |
| *Pseudomonas frederiksbergensis* | Site 1 | rhizosphere | 99.78 | S1C8 | PZ098780 |
| *Pseudomonas iranica* | Site 2 | rhizosphere | 99.14 | S2C12 | PZ098781 |
| *Sinorhizobium kummerowiae*/*meliloti* | Site 2 | rhizosphere | 99.85 | S2C7 | PZ098779 |
